# Nickel‐Based Cocatalysts on Titanium‐Doped Hematite Empower Direct Photoelectrochemical Valorisation of 5‐Hydroxymethylfurfural

**DOI:** 10.1002/cssc.202402604

**Published:** 2025-01-21

**Authors:** Irene Carrai, Raffaello Mazzaro, Caterina Bellatreccia, Alberto Piccioni, Marco Salvi, Silvia Grandi, Stefano Caramori, Paola Ceroni, Luca Pasquini

**Affiliations:** ^1^ Department of Physics and Astronomy University of Bologna Viale Berti Pichat 6/2 40127 Bologna Italy; ^2^ Institute for Microelectronics and Microsystems National Research Council via Gobetti 101 40129 Bologna Italy; ^3^ Department of Chemistry “G. Ciamician” University of Bologna Via Selmi 2 40126 Bologna Italy; ^4^ Department of Chemical and Pharmaceutical Sciences University of Ferrara Via Luigi Borsari 46 44121 Ferrara Italy; ^5^ National Interuniversity Consortium of Materials Science and Technology (INSTM) University of Ferrara Research Unit 44121 Ferrara Italy

**Keywords:** Biomass valorisation, Hematite, Nickel cocatalysts, Photocatalysis, X-ray absorption spectroscopy

## Abstract

The photoelectrochemical oxidation of 5‐hydroxymethylfurfural (HMF), a biomass‐derived intermediate, to 2,5‐furandicarboxylic acid (FDCA), a key building block for industrial applications, is a well‐studied anodic reaction. This photoelectrochemical (PEC) conversion typically requires an electron mediator, such as TEMPO, regardless of the semiconductor used. Various electrocatalysts can also perform this reaction electrochemically, without additional organic species in the electrolyte. In this study, Ti‐doped hematite (Ti:Fe_2_O_3_) photoanodes were employed for the HMF photoelectrochemical conversion at the anodic side of a two‐compartments PEC cell. To avoid the need of an electron mediator, nickel‐based electrocatalysts were deposited on the electrode′s surface. The Ni(OH)_2_‐electrodeposited (Ti:Fe_2_O_3_−Ni) and the NiMo‐sputtered Ti:Fe_2_O_3_ photoanodes (Ti:Fe_2_O_3_−NiMo) were characterised and tested for the HMF oxidation in 0.1 M NaOH (pH 13) electrolyte. Partial HMF photoelectrochemical conversion to FDCA was achieved, pointing out the beneficial effect of Ni‐based cocatalyst in shifting the selectivity towards the di‐carboxylic acid. Fixed Energy X‐ray Absorption Voltammetry (FEXRAV) and X‐ray Absorption Near‐Edge Structure (XANES) measurements were conducted to investigate the interaction between HMF and the two deposited electrocatalysts. These techniques offered valuable insights into the oxidation mechanism, which were further validated using a rate deconvolution procedure.

## Graphical abstract

Nickel modified titanium doped hematite photoanode shows a selective hole scavenging for the photoelectrochemical valorisation of the biomass derivative HMF to FDCA, overcoming the competition with water oxidation. Insights on the role of nickel in the HMF oxidation process are achieved with operando x‐ray absorption spectroscopy via element‐selective voltammetry.

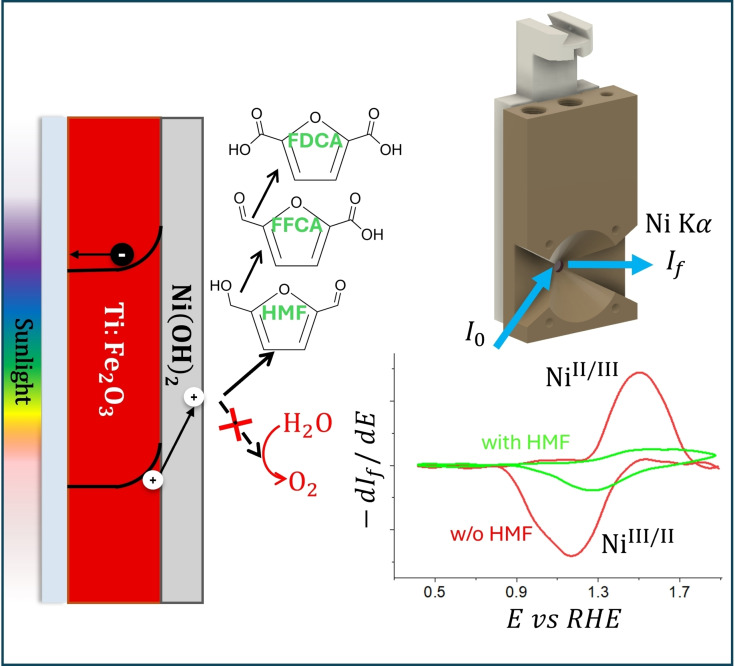

## Introduction

**Scheme 1:**
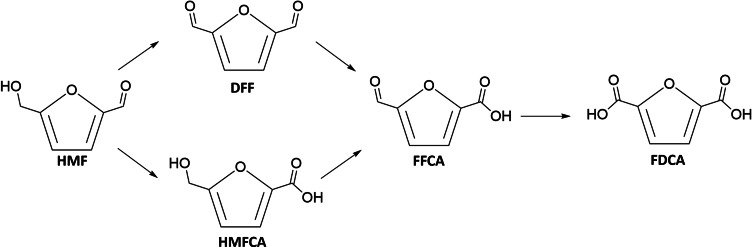
Traditional HMF oxidation mechanism to FDCA. Two possible routes can be followed by HMF: the alcoholic group can be first oxidised to DFF, or the aldehydic group is primarily oxidised to HMFCA. For each of these oxidative steps, 2 electrons are exchanged.

The thermodynamic and kinetic limitations of the oxygen evolution reaction (OER) are pushing the photoelectrochemical (PEC) community to explore more favourable anodic reactions.[^1^, ^2^, ^3^, ^4^] Efforts are underway to upcycle polymeric materials like polyethylene terephthalate (PET) into elemental chemicals such as acetate or formate, which can be used in pharmaceuticals, energy, and food industries.[^5^, ^6^, ^7^, ^8^] Additionally, the oxidation of glycerol, a common by‐product of biodiesel synthesis, into valuable chemicals like dihydroxyacetone (DHA) or glyceric acid (GLA) is being widely investigated.[^9^, ^10^, ^11^, ^12^, ^13^, ^14^] Another promising feedstock valorisation route is the oxidation of the biomass derivative 5‐hydroxymethil furfural (HMF) into 2,5‐furan dicarboxylic acid (FDCA), through the multi‐step pathway schematized in Scheme 1. The latter can be used for the synthesis of polyethylene furanoate (PEF), an environmentally friendly alternative to PET.[^15^, ^16^, ^17^] In principle, the (photo)electrochemical conversion of HMF requires lower activation energy than oxygen evolution from water and, like all the aforementioned oxidation reactions involving organic species, occurs at less anodic potential compared to OER. However, as many oxidative intermediates can be generated from the same starting compound, a deep awareness on the underlying reaction mechanism is necessary. Consequently, the photoanode must be carefully selected, evaluating the material′s stability, performance, and sustainability, in addition to the specific selectivity towards the desired products.

Photoanodes based on BiVO_4_, Fe_2_O_3_ and WO_3_ semiconductors have been previously employed for the PEC oxidation of HMF to FDCA, exhibiting high faradaic efficiency against competing OER.[^18^, ^19^, ^20^] The modification of Ti:Fe_2_O_3_ with Co‐based cocatalysts^[21]^ was also demonstrated useful to further enhance the selectivity towards FDCA. However, in all these examples the addition of an electron mediator, i.e. 2,2,6,6‐Tetramethylpiperidin‐1‐yl)oxyl, (TEMPO), was necessary to promote and control the HMF conversion to FDCA. Indeed, the presence of the radical proved to be essential to scavenge photogenerated holes at the electrode′s surface, accelerating their transfer to the organic species in the electrolyte.[^22^, ^23^] At the same time, hole scavenging by TEMPO limited the formation of oxygen reactive species like OH radicals, formed upon monoelectronic water oxidation, which may trigger uncontrollable oxidation reactions. However, the elevated market price (5–10 $/g) and its potential degradation under reaction conditions, make the use of TEMPO problematic. In addition, once the reaction is complete, a further step must be introduced in the process scale‐up to separate the scavenger from the reaction mixture.

**Scheme 2:**
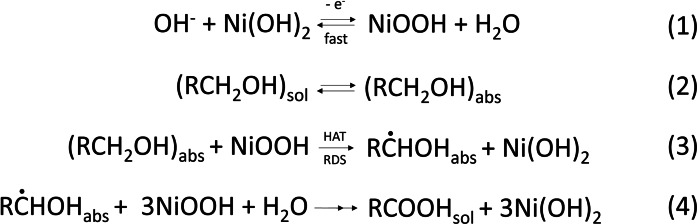
Mechanism for the indirect oxidation of alcohols on NiOOH electrodes in alkaline media, as proposed by Fleischmann et al.[^27^, ^28^] For the aldehydes (RCHO instead of RCH_2_OH), an initial hydration is needed to form the 1,1‐geminal diol, before undergoing the equivalent oxidation mechanism.^[29]^

Direct electrocatalytic HMF oxidation, in absence of redox mediator, was previously achieved by means of electrocatalysts[^24^, ^25^] based on Ni, an affordable transition metal with a low supply risk indicator.^[26]^ Previous works showed that two possible pathways can be followed by nickel‐based catalysts to electrochemically oxidise alcohols and aldehydes. In the indirect oxidation mechanism (Scheme 2), first reported by Fleischmann et al.,[^27^, ^28^] the catalyst is oxidized by the applied potential from Ni^2+^ to Ni^3+^ (process 1), followed by the hydrogen atom transfer (HAT, process 3) from the carbon in the α position of the alcohol (or geminal diol, in the case of the aldehyde) to the Ni^3+^ site, therefore reducing the catalyst back to its initial state. This step is also considered to be the rate determining step (RDS) of the entire process. In this mechanism, both the alcoholic and the aldehydic groups are directly oxidized to the corresponding carboxylic acid and 4 electrons are involved (Scheme 2).

**Scheme 3:**
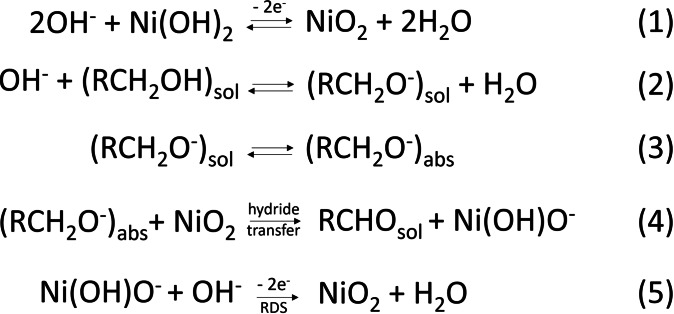
Potential dependent (PD) oxidation mechanism proposed by Bender et al.^[29]^ In this case, the alcohols are oxidised to aldehydes through hydride transfer. Aldehyde oxidation to carboxylic acid can occur according to the same mechanism, prior the establishment of an equilibrium with its hydrated form, i. e., the 1,1‐geminal diol.

At more positive potentials, the so called potential dependent (PD) oxidation mechanism can take place (Scheme 3).^[29]^ In this case, the applied potential is needed not only to oxidize Ni^2+^ to Ni^4+^ (process 1), but also to drive the hydride transfer reaction (process 4), through which the alcoholic group is oxidized to aldehyde (while, for the aldehyde, the 1,1‐ geminal diol is oxidized to carboxylic acid). The RDS is ascribed to the regeneration of the catalyst to its Ni^4+^ state (process 5).

It has been demonstrated that aldehyde groups preferentially oxidise through the indirect pathway, while alcoholic groups follow the PD one. Since HMF has both an alcoholic and aldehydic group in its structure, the dominant mechanism will depend on the experimental conditions applied to perform the oxidation.^[29]^

In this study, to avoid the use of TEMPO, Ti‐doped hematite (Ti:Fe_2_O_3_) photoanodes were modified with nickel‐based cocatalysts and tested for the PEC oxidation of HMF to FDCA at the anodic side of a two‐compartments PEC cell. While the Ni(OH)_2_/NiOOH redox couple has been widely investigated for the electrochemical oxidation of various substrates, only few works report the use of NiMo cocatalyst for anodic reactions, mostly focusing on OER.^[30]^ Since NiMo can also be employed to perform the reductive semi‐reaction at the cathode, e. g., the hydrogen evolution reaction (HER) or CO_2_ reduction,[^31^, ^32^, ^33^] its bifunctional activity will help reducing the fabrication impact of the PEC device, using the same cocatalyst at both electrode sides. Most importantly, with this approach an abundant energy source, such as sunlight, will be exploited to promote HMF oxidation, in absence of redox mediator or critical raw materials‐based cocatalysts.^[26]^ This strategy not only enhances the sustainability of the process but also improves its cost‐effectiveness and scalability, making it a viable pathway for efficient HMF valorisation under eco‐friendly conditions.

The efficiency of the conversion process in alkaline environment (0.1 M NaOH pH 13) was evaluated by combining the photoanode photoelectrochemical characterization with the monitoring of the reaction evolution by High‐Performance Liquid Chromatography (HPLC) and spectro‐photometric/fluorometric techniques. Insights into the reaction mechanism were gained by means of operando electrochemical X‐ray Absorption spectroscopy (XAS), focusing on the role of HMF addition on Ni oxidation state at various selected potentials. In particular, the novel method of Fixed Energy X‐ray Voltammetries (FEXRAV) provided element‐specific voltammetries that highlight the microscopic interaction between Ni and HMF, revealing a remarkable selectivity for the oxidation of such organic substrate. Notably, the insights gained through this technique were corroborated by the rate deconvolution procedure^[29]^ applied to our samples. This combined approach provided a deeper understanding of both the reaction kinetics and the underlying mechanism.

## Results and Discussion

### PEC Characterization

Thin Ti:Fe_2_O_3_ films were deposited following a previously published procedure,^[21]^ summarized in the SI. The resulting film exhibits a thickness ranging between 1.5 and 1.8 μm, and a strongly nanostructured surface (Figure S1). The deposition of Ni‐based cocatalysts was performed according to different techniques: via Ni(OH)_2_ electrodeposition,^[29]^ resulting in Ti:Fe_2_O_3_−Ni photoanode, and via NiMo sputtering, leading to Ti:Fe_2_O_3_−NiMo photoanode. The morphology of the samples was characterized by SEM and reported in Figure S2 and S3. The electrodeposition approach results in the formation of lamellar nanostructures on the Ti:hematite substrate, as typically reported for this method.^[34]^ On the other hand, the sputtering method does not display any directional microstructure due to the continuous rotation of the sample during the deposition process.

**Figure 1:**
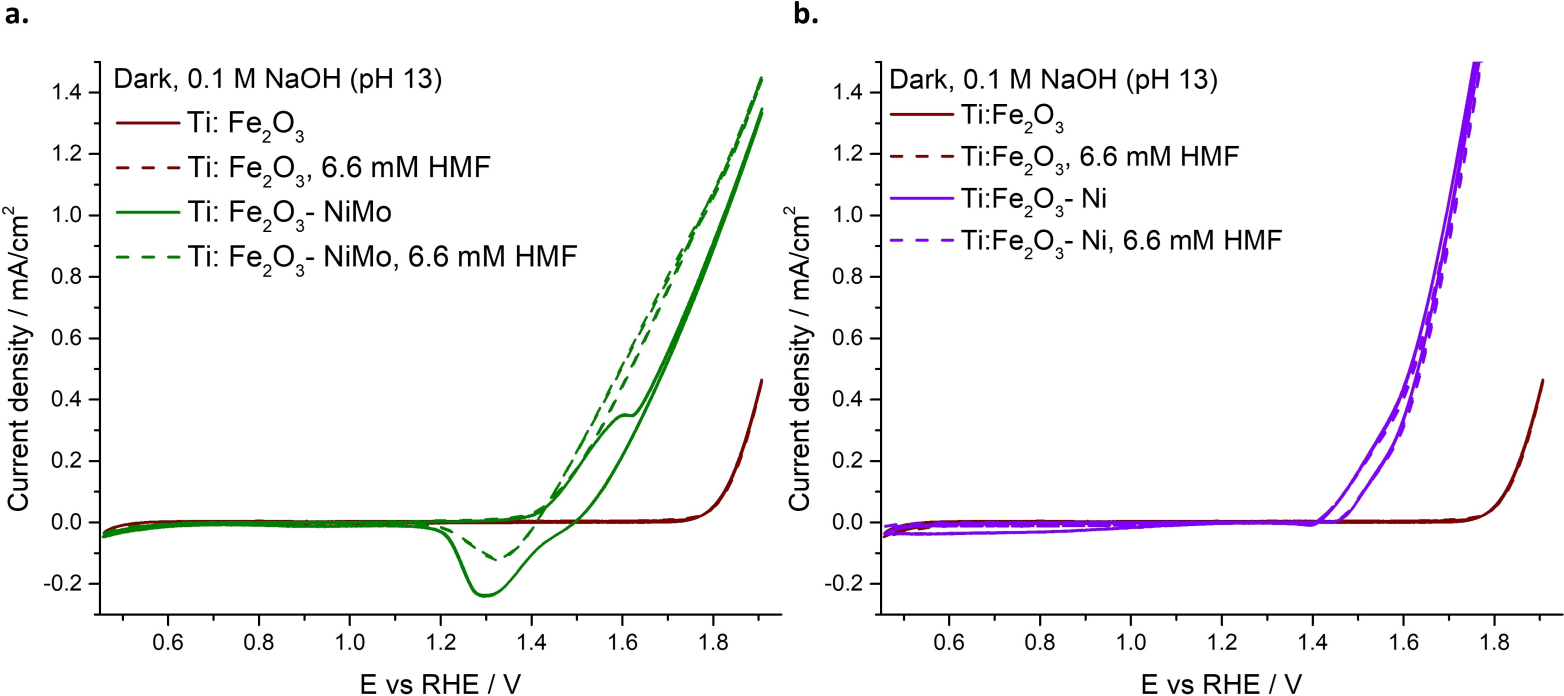
Dark CVs before (solid line) and after (dotted line) the addition of HMF with: (a) Ti:Fe_2_O_3_−NiMo (green) and Ti:Fe_2_O_3_ (red); (b) Ti:Fe_2_O_3_−Ni (violet) and Ti:Fe_2_O_3_ (red) photoanodes.

The electro‐ and photoelectro‐chemical activity of the synthesized Ni‐modified Ti‐doped hematite photoanodes, i. e., Ti:Fe_2_O_3_−Ni and Ti:Fe_2_O_3_−NiMo, was tested and compared with the bare Ti:Fe_2_O_3_. The dark CVs acquired for the Ni‐modified photoanodes highlight a significant anticipation of the dark onset potential compared to Ti:Fe_2_O_3_ (Figure 1). In the electrolyte without HMF, Ti:Fe_2_O_3_−NiMo exhibits the oxidation/reduction waves usually attributed to the Ni(OH)_2_/NiOOH redox couple^[29]^ (Figure 1a, solid line). The intensity of these waves is linearly dependent on the scan rate, as also observed with NiMo sputtered on FTO (Figure S4), showing a behaviour typical of surface processes.^[35]^ When HMF is introduced (Figure 1a, dashed line), the oxidation wave disappears, indicating a rapid transfer of holes to the organic substrate. As a result, the reduction wave significantly decreases, suggesting that most of the catalyst has already been reduced by the swift transfer of positive charge to HMF.

For Ti:Fe_2_O_3_−Ni photoanode in the electrolyte without HMF, the oxidation/reduction peaks between 1.2 V‐1.4 V vs RHE, typically attributed to the Ni(OH)_2_/NiOOH redox couple,^[29]^ are barely noticeable (Figure 1b, solid line). Additionally, a broad feature appears in the reverse scan, extending across a wide region of cathodic potentials. Notably, this reductive wave is unique to this photoanode, as it was not observed with either Ti:Fe_2_O_3_ or Ti:Fe_2_O_3_−NiMo electrodes. Further measurements confirm that this behavior is intrinsic to the electrodeposited Ni(OH)_2_, as it occurs also with Ni(OH)_2_ deposited on FTO and persists when CVs are conducted under Ar (Figure S5). Upon addition of HMF, the feature in the cathodic region disappears (Figure 1b, dashed line). Thus, as in the case of Ti:Fe_2_O_3_−NiMo, the addition of the organic substrate would produce a swift scavenging of the excess positive charge on the oxidized Ni site, preventing the cathodic process related to the cocatalyst reduction.

**Figure 2:**
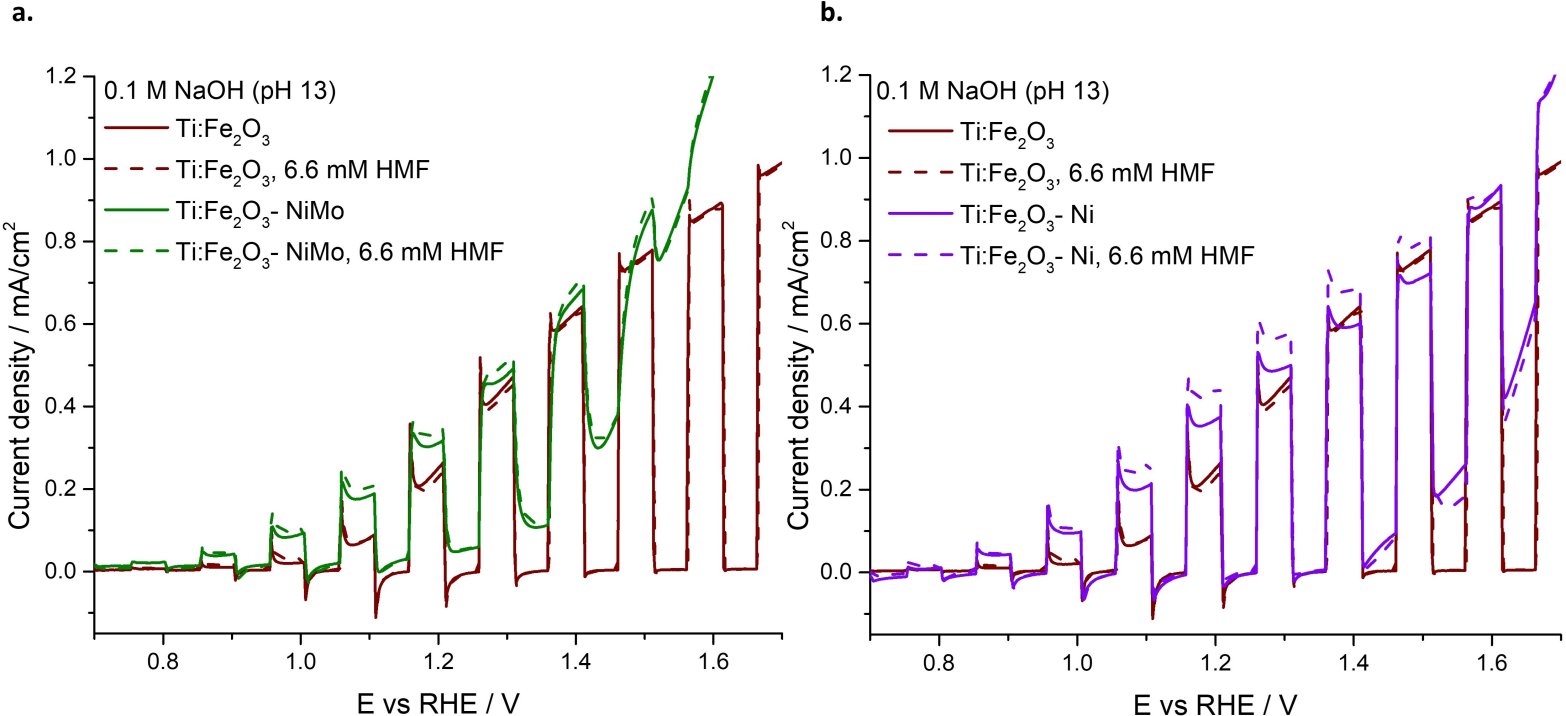
Chopped LSVs before (solid lines) and after (dashed lines) the addition of HMF for: (a) Ti:Fe_2_O_3_−NiMo (green) and (b) Ti:Fe_2_O_3_−Ni (violet) photoanodes, Ti:Fe_2_O_3_ (red) reported for comparison.

Chopped linear sweep voltammetries (LSVs) were collected for the three photoanodes, before (solid lines) and after (dashed lines) the addition of HMF to the electrolyte (Figure 2).

The presence of the electrocatalysts anticipates both the dark‐ and the photo‐ onset potentials and reduces the recombination spikes observed with Ti:Fe_2_O_3_ photoanode (red lines). As a result, Ti:Fe_2_O_3_−Ni and Ti:Fe_2_O_3_−NiMo exhibit a sizeable photocurrent density before the water oxidation potential, with a further increase upon the addition of HMF, specifically with Ti:Fe_2_O_3_−Ni photoanode. This observation suggests a high selectivity towards HMF oxidation, consistent with the dark measurements (Figure 1).

The PEC stability of the Ni‐modified Ti:Fe_2_O_3_ photoanodes was assessed by steady‐state chronoamperometry at 1.4 V vs RHE (Figure S6). For all the samples, after an initial decrease of the current density, the values remain stable during the whole experiment with only little changes when HMF is added to the electrolyte. Notably, the photoanodes used in this study demonstrated an excellent stability during the long‐term photoelectrolysis (Figure S7 and S8), and they could be used multiple times without requiring redeposition of the Ni‐based electrocatalysts (Figure S9). Ti:Fe_2_O_3_−NiMo undergoes a severe change of the surface morphology during PEC operation, attributed to the formation of a hydroxide phase (Figure S2) and later confirmed by X‐ray Absorption Spectroscopy. Limited modification is observed on Ti:Fe_2_O_3_−Ni, where the lamellar structure is already visible in pristine sample. For both samples, it is worth noting how the lamellar structures form deep within the Ti:Fe_2_O_3_ layer (Figure S3), facilitated by the presence of diffused cracks in the film. However, the intrinsic porosity of the lamellar film may lead to partial exposure of the underlying hematite layer. The resulting distribution of exposed sites, either coated with the Ni‐based overlayer or pristine hematite, is likely producing an averaged contribution on the electrochemical activity.

### HMF PEC Conversion

**Figure 3:**
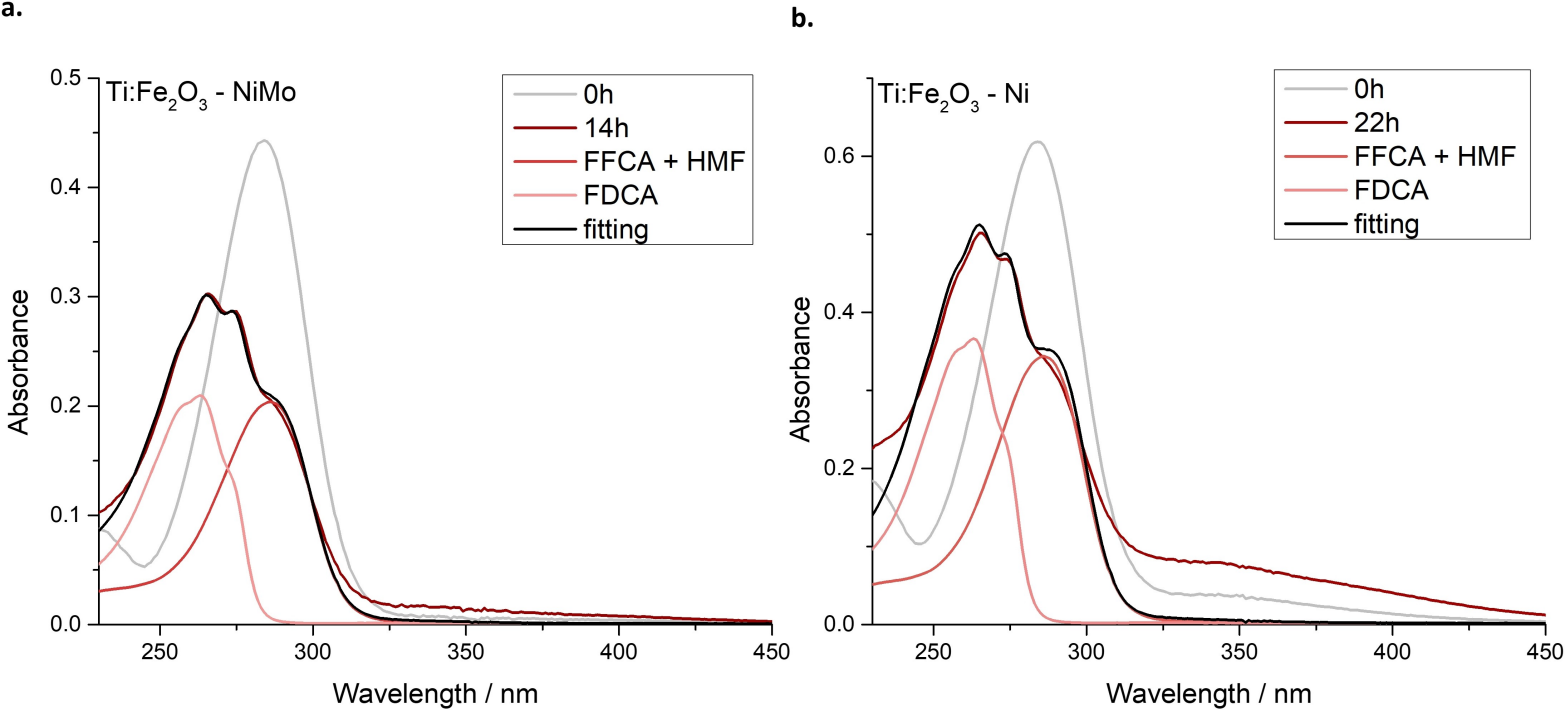
Evolution of the absorption spectra after long‐term PEC HMF conversion: the initial (0 h, grey line) and final spectra (dark red line) are compared to reference FFCA or HMF (red line) and FDCA (light red line). The black line represents the best fit function. (a) Ti:Fe_2_O_3_−NiMo and (b) Ti:Fe_2_O_3_−Ni photoanodes.

As discussed in the previous section, Ni and NiMo cocatalysts significantly influence the photoelectrochemical behaviour of Ti:Fe_2_O_3_ photoanode, enabling a selective hole scavenging before the onset of OER (Figure 2). The results of the long‐term PEC conversion of HMF to FDCA are summarized in Figure 3 and Table 1.

**Table 1:**
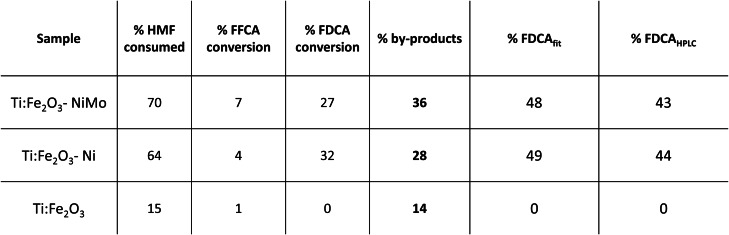
Outcomes of the long‐term PEC HMF conversion for the bare and Ni‐modified Ti:Fe_2_O_3_ photoanodes: percentage of HMF consumed, from which the percentages of FFCA, FDCA and by‐products were calculated. The relative coefficient (%) for FDCA, determined both from the fitting and the HPLC data, are also illustrated.

For both photoanodes, the initial HMF absorption spectrum (grey line in Figure 3) mutates to a structured spectrum (dark red lines), where a maximum around 263 nm suggests the evolution of the reaction towards the desired product FDCA, given the spectral matching with reference FDCA (Figure S10). It is worth noting the presence of a shoulder within 320–450 nm, which cannot be attributed to HMF oxidative intermediates, but rather to possible by‐products. These species are responsible for the yellowish colour of the solution and fluoresce upon excitation at λ=350 nm (Figure S11). Since the colour of the solution darkened over time, the higher shoulder visible for Ti:Fe_2_O_3_−Ni can be ascribed to the longer duration of the experiment: 22 h instead of 14 h to reach the same final charge as Ti:Fe_2_O_3_−NiMo. The black lines in Figure 3 represent the best fit of the final spectra by a combination of reference spectra, from which the percentage of FDCA (%) in Table 1 was determined (more information about the fitting function are reported in the SI).

By means of High‐Performance Liquid Chromatography (HPLC), a more precise quantitative evaluation of the conversion percentage was carried out. As summarized in Table 1 and in agreement with the spectro‐photometric analysis, although the HMF to FDCA conversion was substantial, a complete conversion was still not achieved, and a large quantity of by‐products was also detected. The occurrence of these side reactions, and the potential strategies to mitigate them, will be better addressed in the following paragraphs. HMFCA and DFF (Scheme 1) were not detected and the FDCA conversion was similar for the two photoanodes: 27 % for Ti:Fe_2_O_3_−NiMo and 32 % for Ti:Fe_2_O_3_−Ni. Notably, when bare Ti:Fe_2_O_3_ was employed (Figure S12), only a small amount of HMF was consumed, with the conversion predominantly following undesired pathways to by‐products, pointing out the pivotal role of the Ni‐based cocatalyst in driving the reaction towards a comparatively much more efficient and selective HMF oxidation.

Finally, the faradaic efficiencies *η_F_* were determined according to the following Equation:

(1) $$\;\eta_{F}=\frac{mol\;of\;product\;formed}{Q_{f}/\left(F\cdot n\right)}x\;100$$

where the *mol of product formed* were calculated from the HPLC results, *Q_f_* is the total charge passed at the end of the experiment, i. e. 57 C, *F* is the Faradaic constant 96486 C mol^−1^ and *n* are the number of electrons exchanged for a specific oxidative step. The resulting faradaic efficiency for FDCA are 42 % for Ti:Fe_2_O_3_−NiMo and 30 % for Ti:Fe_2_O_3_−Ni, while it is 0 for the bare Ti:Fe_2_O_3_, emphasizing the crucial role of Ni‐based cocatalysts in achieving precise and selective oxidation of the organic substrate. The relatively limited *η_F_* for Ti:Fe_2_O_3_−NiMo and Ti:Fe_2_O_3_−Ni is reflecting the distribution of exposed sites, where the residual exposed hematite sites may hinder the beneficial role of the Ni‐based overlayer on the faradaic efficiency towards HMF oxidation.

Table 1 also shows that the relative amount of FDCA (excluding by‐products), determined by fitting the optical spectra (% FDCA_fit_, Equation S2 in the SI), aligns well with the relative conversion coefficient obtained from the HPLC results (% FDCA_HPLC_, Equation S3 in the SI), despite a slight overestimation. This emphasizes that, while the HPLC analysis is essential for an accurate quantitative evaluation, spectro‐photometry can still provide an effective marker of the produced FDCA, although it is not possible to differentiate between HMF and FFCA given the similarity of their reference spectra (Figure S10).

To investigate the nature of the by‐products (Table 1) as well as to understand the role of the cocatalysts in the HMF PEC conversion, further experiments were performed, the full details of which are reported in the SI. The results reveal the dissolution of Mo in basic aqueous environment (Figure S13 and S14), justifying the electrochemical behaviour of Ti:Fe_2_O_3_−NiMo photoanode, for which the contribution of molybdenum was absent in both oxidation and reduction scans of the dark CVs (Figure 1a). According to data from a previous study,^[30]^ Mo dissolution from NiMo at high pH levels does not hinder, but rather enhances, cocatalyst performance. Indeed, it leads to an increase in the specific surface area, enriching the Ni sites that are catalytically active for HMF oxidation. HMF instability in highly alkaline solutions was also observed (Figure S15), explaining the presence of unidentified by‐products during prolonged photoelectrolysis experiments. However, ensuring a high concentration of OH^−^ in the electrolyte is essential for efficient HMF PEC oxidation in the presence of Ni‐based cocatalysts.^[25]^ This was further confirmed by photoelectrolysis experiments performed under the same conditions but at milder pH levels, which showed no conversion of HMF (Figure S16).

Overall, with both Ti:Fe_2_O_3_−Ni and Ti:Fe_2_O_3_−NiMo photoanodes, HMF PEC oxidation to FDCA was achieved with good yields. It is known that, by working in highly alkaline solutions, two possible pathways can be followed by HMF: it can either degrade to humins, oligomers very difficult to detect with the common product analysis techniques, such as HPLC or NMR,^[36]^ or it can undergo the Cannizzaro reaction converting into DHMF or HMFCA which can still electrochemically oxidise to the desired product, FDCA. Depending on the base and substrate concentrations, as well as on the reaction temperatures, HMF will preferentially decompose according to one of these two routes.[^37^, ^38^] Usually, high temperatures, low base and substrate concentrations cause the HMF degradation to humins. Thus, if part of the starting compound is involved in this side‐reaction, a reduced amount will be available to interact with nickel‐based cocatalysts and evolve through the PEC process to the FDCA product. In the future, the adjustment of the above parameters could enable the optimisation of the synthetic procedure towards the Cannizzaro compounds.^[37]^ In this way, the good hole scavenging performed by HMF on nickel‐modified Ti‐doped hematite photoanodes will be effectively exploited to get FDCA.

### X‐ray Spectro‐Electrochemical Analysis

**Figure 4:**
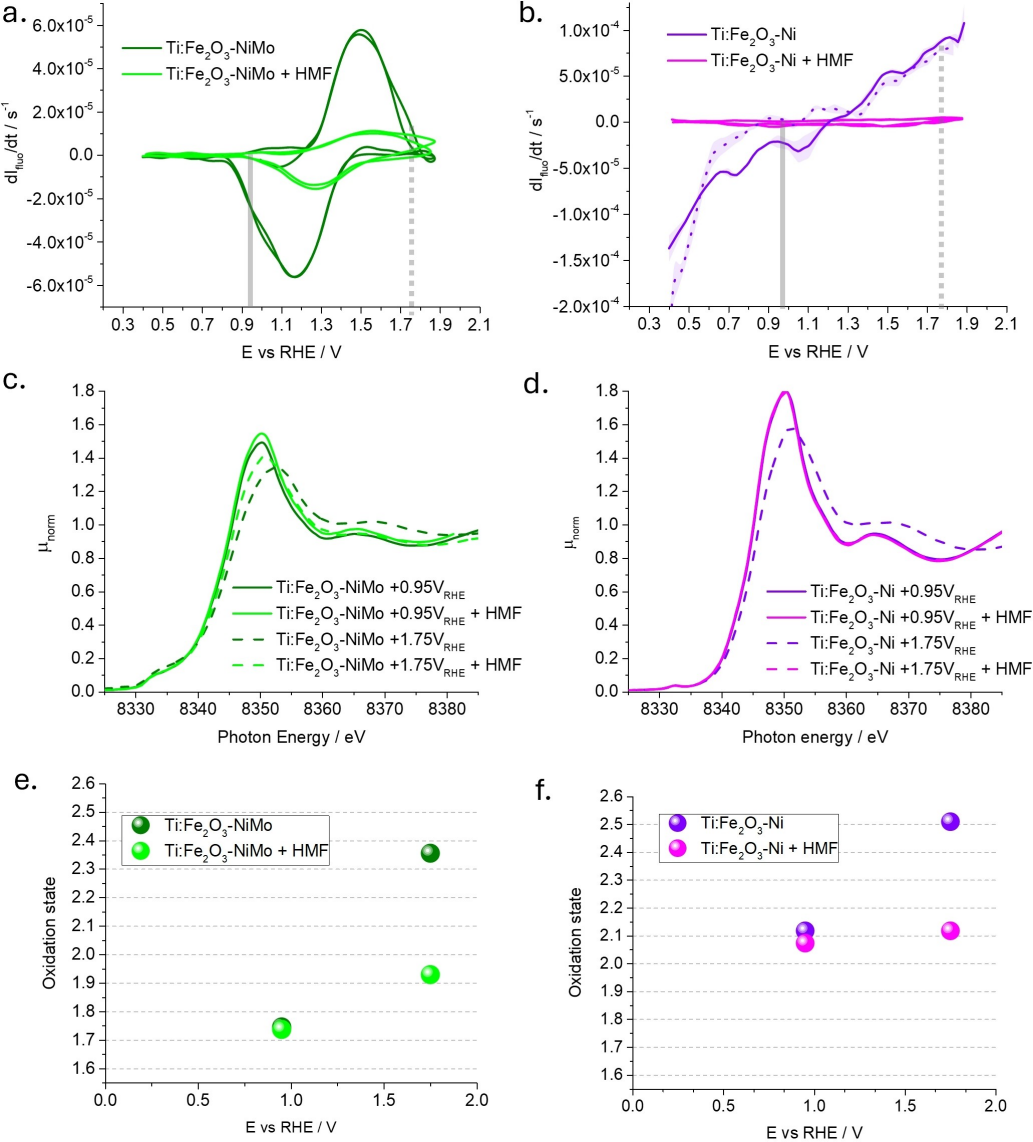
X‐ray spectro‐electrochemical characterization of Ti:Fe_2_O_3_−NiMo (green) and Ti:Fe_2_O_3_−Ni (violet) photoanodes before (solid line) and after (dotted line) the addition of 6.6 mM HMF to 0.1 M NaOH electrolyte (pH 13). (a, b) Derivative of the FEXRAV signal registered at fluorescence energy equal to 8345 eV, before and after HMF addition. The anodic sweep in Fig. b is reported as a dotted line to highlight the hysteresis with the cathodic sweep. (c, d) XANES Ni K‐edge spectra at selected potentials, highlighted in the FEXRAV analysis as solid and dashed grey markers. (e, f) Corresponding oxidation states obtained by calibration with standards, applying the integral method for a reliable definition of the absorption edge energy.

Fixed‐energy X‐ray Absorption Voltammetry (FEXRAV) experiments for Ti:Fe_2_O_3_−NiMo and Ti:Fe_2_O_3_−Ni were carried out to monitor the Ni K‐edge chemical shift *operando* as a function of the applied potential.^[39]^ More details on the experimental setup and technique are available in the experimental section and in the SI (Figure S17). Figure S18 reports the variation of the normalized Ni Kα fluorescence I_fluo_ (proportional to the x‐ray absorption coefficient μ) upon potential sweeping and HMF addition. The derivative ‐dI_fluo_/dt as a function of the potential is reported in Figure 4a and b, respectively for Ti:Fe_2_O_3_−NiMo and Ti:Fe_2_O_3_−Ni.

By comparing the voltammetry for Ti:Fe_2_O_3_−NiMo in the absence of HMF and illumination (Figure 1a, dotted line) to the x‐ray spectro‐electrochemical FEXRAV plot in Figure 4a, we can recognize the reversible Ni ox/red wave, centred at E_eq_=+1.4 V vs RHE. Unlike the conventional voltammetry, FEXRAV provides an element‐selective information on the potential‐dependent variation of the oxidation state of the cocatalyst, complementary to the information provided by conventional voltammetry.^[40]^ Indeed, upon addition of HMF, the wave strongly dampens in intensity, pointing out that a smaller variation of Ni oxidation state occurs in this case, due to quenching of the oxidized Ni states by HMF acting as an electron donor. This is to be contrasted with conventional voltammetry, where the same mechanism, i. e. electrochemical oxidation of HMF at the expense of Ni, leads to a catalytic amplification of the anodic current. This points out that HMF acts as a hole scavenger for the NiMo layer. A similar behavior is observed on Ti:Fe_2_O_3_−Ni, despite the broadened waves and the lack of a sharp reversible wave for this photoanode.

Further, XANES spectra were measured at selected potentials of +0.95 V and +1.75 V vs RHE, to confirm the chemical shift on the absorption edge: these are reported in Figure 4c–d for Ti:Fe_2_O_3_−NiMo and Ti:Fe_2_O_3_−Ni with and without HMF addition. For both samples, at +0.95 V vs RHE the XANES spectra are unaltered after the addition of HMF, pointing out that no spontaneous process occurs in this potential region. Figure 4e–f display the nominal oxidation state determined from the x‐ray absorption edge energy by means of the integral method and calibration curve, as explained in the SI (Figure S19). The oxidation state of the Ni site is Ni^1.75+^ and Ni^2.12+^ for NiMo and Ni electrocatalysts, respectively. Thus, Ni is oxidised when being exposed to the electrolyte, and the extent of the oxidation process is slightly lower when Ni is coupled to Mo, suggesting a partial polarization of electron density from molybdenum to nickel. When the potential is set to +1.75 V vs RHE in the Ti:Fe_2_O_3_−NiMo sample, the spectrum is blue‐shifted due to the chemical shift induced by the electrochemical oxidation process. The corresponding oxidation state is increased to Ni^2.35+^ and Ni^2.52+^ for Ti:Fe_2_O_3_−NiMo and Ti:Fe_2_O_3_−Ni, respectively. Upon addition of HMF, the process is either partially or fully reversed, with the oxidation state being reduced to Ni^1.92+^ and Ni^2.12+^, respectively, highlighting the strong selectivity of these photoanodes towards HMF oxidation. In the steady state at +1.75 V, the holes stored in the Ni cocatalyst layer are swiftly scavenged by HMF, resulting in a return of Ni to the low oxidation state. This finding corroborates the suppression of the reductive peak observed in the dark CVs for both Ti:Fe_2_O_3_−NiMo and Ti:Fe_2_O_3_−Ni photoanodes (Figure 1a and 1b). Notably, this prompt transfer of holes occurs at potentials where a strong competition with water oxidation is expected. Therefore, when Ni‐based electrocatalyst are present on the photoanode′s surface, the HMF′s holes scavenging capability is maximized. This prevents the need for an electron mediator to facilitate HMF oxidation and mitigates issues associated with competition from water oxidation.

Finally, we would like to point out that the oxidation state from XAS measurements is determined by averaging across all the Ni atoms present on the sample′s surface. Therefore, this knowledge alone cannot be used to define the reaction mechanism in a dynamic fashion. Additionally, HPLC data were collected only at the beginning and at the end of the experiment, providing no information about intermediate products. However, the rapid charge transfer between Ni‐based catalysts and HMF, revealed by operando XAS, suggests that the RDS is not related to charge transfer steps between the electrocatalysts and HMF (Scheme 2, process 3 and Scheme 3, process 4). A comprehensive mechanistic analysis is presented in the following section.

### Mechanistic Discussion

To unravel the mechanistic details of the catalytic cycle in the Ti:Fe_2_O_3_/Ni‐cocatalyst system, we performed a kinetic analysis using an electroanalytical method adapted from the approach by Bender and Choi.^[29]^ This three‐step rate deconvolution procedure enables a detailed examination of electron transfer processes under realistic operational conditions.

Briefly, this method begins with a pre‐conditioning step where nickel is oxidised from Ni(OH)_2_ to NiOOH at the potential used during electrolysis experiments. To account for the role of photoinduced carriers, this step was modified and conducted under simulated sunlight (see SI). In the second step, the system was held at open‐circuit potential (OCP) for a specific time, during which the PD mechanism was inactive (Scheme 3). However, NiOOH could still react with HMF through the indirect mechanism (Scheme 2), reducing back to Ni(OH)₂ while oxidizing the organic substrate. In the third step, a reducing potential was applied to convert any remaining unreacted NiOOH back to Ni(OH)₂. This three‐step cycle was repeated with varying OCP durations, and a plot was generated to track the charge decay in the NiOOH film due to its reaction with HMF over time at OCP. By applying a linear fit to the data, the reaction order (0th, 1st, or 2nd) was determined, along with the relative contributions of the PD and indirect mechanisms (see SI for calculation details).

**Figure 5:**
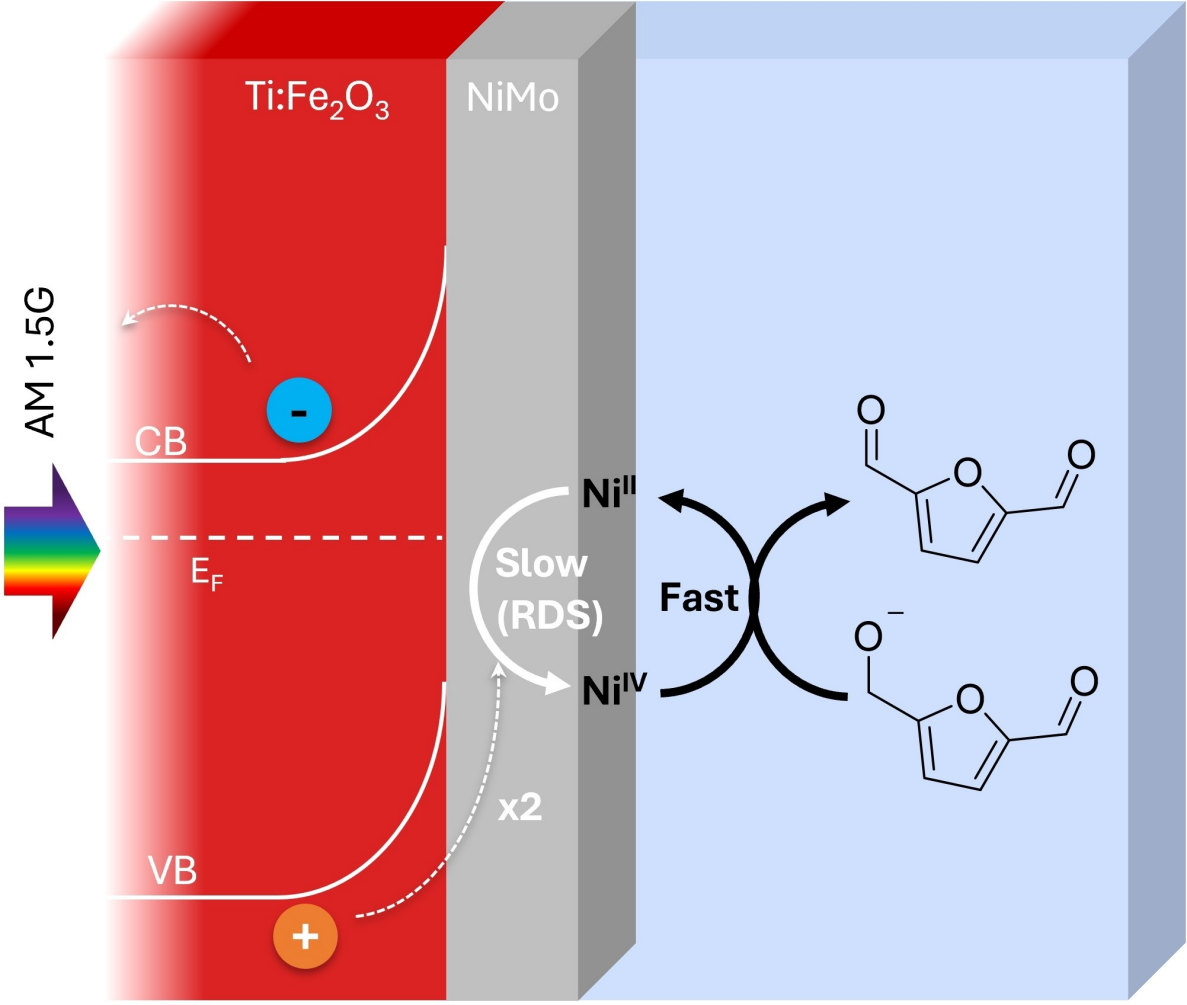
Pictorial representation of the HMF PEC oxidation dynamics by Ti:Fe_2_O_3_ photoanodes coated with Ni‐based cocatalysts.

As shown in Figure S20, this method was applied to Ti:Fe_2_O_3_−NiMo and compared with a reference NiMo on FTO, with both samples exhibiting first‐order kinetics (Equation S4). Table S1 reveals that, under PEC experimental conditions, the PD mechanism dominates over the indirect mechanism, consistent with the findings of Bender et al. for dark electrolysis.^[29]^ In the PD mechanism, the RDS is identified as the regeneration of Ni⁴^+^ following the oxidation of the organic species^[41]^ (process 5 in Scheme 3). This conclusion agrees with our operando XAS measurements, where an immediate decrease in the nickel oxidation state was observed upon the addition of HMF (Figure 4). This suggests that Ni⁴^+^, once formed, is rapidly reduced through interaction with the organic substrate. Consequently, the RDS is not the direct chemical interaction between Ni⁴^+^ and HMF but the regeneration of Ni⁴^+^ from its reduced state, as expected in the PD mechanism (Figure 5 and Scheme 3).

## Conclusions

Nickel‐modified titanium doped hematite photoanodes, i. e. Ti:Fe_2_O_3_−NiMo and Ti:Fe_2_O_3_−Ni, were employed for the first time in the photoelectrochemical oxidation of HMF to FDCA, without the need for additional electron mediator. In a previous work, we showed that such results are unattainable using the same Ti:Fe_2_O_3_ modified with cobalt‐based cocatalysts (e. g., CoPi or CoFeO_x_).^[21]^ Comprehensive photoelectrochemical characterization and operando XAS measurements highlighted the remarkable selectivity of these photoanodes for HMF oxidation over water oxidation, attributed to a strong hole scavenging effect. The use of a rate deconvolution procedure, adapted from previous studies, allowed for a deep understanding of the catalytic activity of nickel toward HMF oxidation, emphasizing the dominant potential‐dependent mechanism and corroborating XAS results. Under the alkaline conditions employed, the photoelectrochemical oxidation to FDCA was incomplete, resulting in the formation of by‐products and highlighting the necessity for further optimization of the reaction parameters. Nevertheless, the proposed system effectively addresses the inherent challenges associated with the inclusion of a redox mediator, enhancing both the reliability and sustainability of the process. This advancement demonstrates significant potential for the valorisation of HMF in photoelectrochemical applications.

## Conflict of Interests

The authors declare no conflict of interest.

1

## Supporting information

As a service to our authors and readers, this journal provides supporting information supplied by the authors. Such materials are peer reviewed and may be re‐organized for online delivery, but are not copy‐edited or typeset. Technical support issues arising from supporting information (other than missing files) should be addressed to the authors.

Supporting Information

## Data Availability

The data that support the findings of this study are available on request from the corresponding author. The data are not publicly available due to privacy or ethical restrictions.
