# Supplementary material for: Nickel‐Based Cocatalysts on Titanium‐Doped Hematite Empower Direct Photoelectrochemical Valorisation of 5‐Hydroxymethylfurfural
Source: ChemSusChem. 2025 Jan 21;18(9):e202402604. doi: 10.1002/cssc.202402604 (PMC12051249; doi:10.1002/cssc.202402604)
Supplement: Supplementary file 1 — Supporting Information [file CSSC-18-e202402604-s001.pdf]

ChemSusChem

Supporting Information

Nickel-Based Cocatalysts on Titanium-Doped Hematite Empower Direct Photoelectrochemical Valorisation of 5-Hydroxymethylfurfural

Irene Carrai, Raffaello Mazzaro,* Caterina Bellatreccia, Alberto Piccioni, Marco Salvi, Silvia Grandi, Stefano Caramori, Paola Ceroni, and Luca Pasquini*

Nickel-based cocatalysts on Titanium-doped Hematite empower direct photoelectrochemical valorisation of 5-Hydroxymethylfurfural

Irene Carrai, Raffaello Mazzaro*, Caterina Bellatreccia, Alberto Piccioni, Marco Salvi, Silvia Grandi, Stefano Caramori, Paola Ceroni, Luca Pasquini*

*Corresponding Authors, e-mail: raffaello.mazzaro@unibo.it; luca.pasquini@unibo.it

Experimental Details

Materials. Chemicals and solvents were purchased from Merck, Alfa Aesar and Carlo Erba and were used as received. A MilliporeTM system was exploited to produce deionized water employed for material preparations.

Ti-doped hematite photoanodes preparation. The photoanode fabrication and structural characterization was made by adapting and combining previously reported synthetic approaches[1],[2]. Ti (IV) doped nanostructured hematite photoanodes (Ti:Fe_2O_3) were prepared on a fluorine doped SnO_2 (FTO) conductive glass by a hydrothermal approach. Briefly, the FTO glass was cleaned through ultrasonication in isopropanol, and then rinsed with deionized water. The fabrication of hematite nano-rod electrodes involves the deposition of a Ti (IV) doped iron oxide seed layer by dip coating (0.625 mm/s) the cleaned 2 mm thick FTO/glass slides (10 mm (wide) x 25 mm (long)) in a Fe (III) oleate precursor containing 15 mM Titanium (IV) isopropoxide to obtain a 10 mm x 10 mm coated area. The dip coating solution was prepared following the procedure described by D. K. Bora[1]. The Fe(III)oleate layer was converted into hematite following a 30 minute heat treatment at 500 °C. Solvothermal synthesis was carried out in a teflon-lined stainless steel autoclave by using an aqueous precursor containing 0.91 M sodium nitrate ($NaNO_3$, Carlo Erba Reagents), at a pH value of 1.5 adjusted with 6 M HCl, 0.136 M of ferric chloride ($FeCl_3 \cdot 6$ H2O, Alfa Aesar), 2.5 mM Ti_2CN (Sigma-Aldrich) and a 5 % (v/v) ethanol (Carlo Erba Reagents)[3]. The seed-layered electrodes were inserted into the autoclave, lying at an angle of ca. 45° with respect to the vertical liner walls. Heating at 95 °C was applied for 4 h. A uniform layer of yellowish colour film (FeOOH) was formed on the electrodes. The FeOOH-coated substrates were washed with deionized water to remove weakly interacting residues from the hydrothermal bath before sintering in air at 550 °C for 1 h, during which conversion of FeOOH to Fe_2O_3 occurred. Finally, the resulting hematite thin films were modified by chemical bath treatment in a 0.2 M $TiCl_4$ solution heated at 50 °C for 1 hour, followed by a final thermal annealing at 760 °C for 10 minutes affording the Ti:Fe_2O_3 electrodes used for this study.

Deposition of nickel-based electrocatalysts on Ti-doped hematite photoanodes and FTO substrates. *$Ni(OH)_2$ electrodeposition:* $Ni(OH)_2$ film was electrodeposited on Ti:Fe_2O_3 photoanode following the procedure of Bender et al.[4] The process was monitored by a Gamry Interface 1010E potentiostat and conducted with a 3-electrodes setup. The working electrode (WE) was Ti:Fe_2O_3 photoanode, the counter electrode (CE) was a platinum wire (Pt), and the reference electrode (RE) was Hg/HgO (1 M NaOH). During the deposition, a current density of -0.25 mA/cm^2 was maintained for 12 min in a 50 mM Ni $(NO_3)_2 \cdot 6H_2O$ solution. *NiMo sputtering:* the NiMo catalyst layer was deposited by DC magnetron sputtering using a 2 inches NiMo target (EVOCHEM ADVANCED MATERIALS) with 80:20 at% composition. The chamber started from a pressure of $5x10^{-7}$ mbar, and we added 10 sccm of Ar to bring the pressure to $8x10^{-3}$ mbar. We sputtered for 20 minutes at 20 W. The sputtering rate for these parameters had been previously calibrated and the film thickness was estimated to be 32 nm.

PEC characterization. Ti:Fe_2O_3-NiMo, Ti:Fe_2O_3-Ni and Ti:Fe_2O_3 photoanodes were characterized in a single compartment, custom-built PEEK cell (Vol= 20 mL) under flow conditions. Since both the semiconductor and

the cocatalysts are stable in basic environment at the working anodic potentials, a 0.1 M NaOH solution (pH 13) was selected as the electrolyte. Hg/HgO (NaOH 1 M) was employed as the reference electrode and Pt as the counter electrode. When needed, 6.6 mM HMF was added to the electrolyte. Chopped LSVs, CVs and CA were performed using a Gamry Interface 1010E potentiostat. The measurements with light were acquired by applying AM 1.5G illumination (100 mW·cm^{-2}) on the backside of the photoanodes. Chopped LSVs were conducted by sweeping the potential to the positive direction at a scan rate of 5 mV/s (step size: 2 mV), at room temperature and without stirring. CVs were performed in the same conditions, by first scanning the potential to the positive direction. When specified, the scan rate was switched to 50, 20 or 10 mV/s (step size: 2 mV). For the measurement at the steady state, a continuous CA was performed for 1h at 1.4 V vs RHE. Although the working-electrode potentials were measured against an Hg/HgO (NaOH 1 M) reference electrode, all the results are reported against the reference hydrogen electrode (RHE). In this way, it is easier the comparison with H_2O/O_2 redox levels as well as with other reports using different electrolytes and pHs. To do this conversion, Nernst Equation was applied:

$$E\ (vs\ RHE)\ =\ E\ (vs\ Hg/HgO)\ +\ E_{Hg/HgO}\ +\ 0.059\ \cdot\ pH\quad (\mathrm{S1})$$

where $E_{Hg/HgO}\ \ (reference) =\ 0.140\ V\ vs\ NHE$ at 25°C.

HMF PEC conversion. The photoelectrochemical conversion experiments were performed in a two-compartment PEEK cell, using 6.6 mM of HMF in 0.1 M NaOH as the electrolyte (pH 13). The two compartments of the cells were separated by a Nafion membrane (N-117, thickness 0.007 in.). A long-term chronoamperometry (CA) was run at 1.4 V vs RHE, under a constant simulated 1 sun-equivalent illumination (Seoul semiconductors Sunlike LED), until a charge equal to 57 C has passed through the electrode. Since the voltage modulation in the steady state has demonstrated to have a beneficial effect on the Ti:Fe_2O_3 photoanodes stability a pulsed working mode was applied to the CA: a potential of 1.4 V vs RHE was maintained for 60s, followed by a cathodic pulse at 0 V vs RHE for 5s; this on/off cycle was repeated until the end of the measurement. To monitor the reaction, the solution was sampled over time. The samples were then analysed, to quantify the oxidation products and to calculate their respective conversion percentages % and faradic efficiencies η_F.

Spectro-photometric analysis. UV-visible absorbance spectra were recorded with a Perkin Elmer λ650 spectrophotometer, using quartz cells with a 1.0 cm path length. The estimated experimental error is 5% on the molar absorption coefficients.

Emission spectra were recorded with an Edinburgh FS5 with a PMT980 and an InGaAs detector for visible and NIR spectral range, respectively.

High Performance Liquid Chromatography (HPLC) analysis. For a more quantitative investigation, HPLC chromatographs were obtained with an HPLC analitica Agilent Technologies 1100 series with a UV-Vis DAD and an isocratic elution with aqueous ammonium acetate (5 mM): MeOH (ratio 7:3), using a reverse phase 25 cm Luna Omega C-18 column. The flow rate was 0.5 mL/min. The injected volume was 2 microliters of the sample diluted 10 times in the eluent phase. Identification and computation of the concentration of the compounds were obtained by calibration curves on chromatograph peaks (from the absorption recorded by diode-array detectors at 254 nm) using solutions of known concentrations (ranging from 0.01 mM to 1 mM) of commercial standards. The retention times were: 4.17 min for FDCA, 5.35 min for FFCA, 8.36 min for HMF and 9.64 min for DFF.

Structural analysis. SEM analysis was conducted with Zeiss LEO 1530 FEG-SEM, equipped with in-lens SE detector for imagine, operated at 5kV. Elemental analysis was performed by Energy Dispersive X-ray (EDX) spectrometer, operating at 20kV. X-Ray Photoelectron Spectroscopy (XPS) measurements utilized a SPECS Focus 500 monochromator and Phoibos 100 electron analyser, using Al Kα radiation at 1486.74 eV. The XPS spectra were measured at a 90° photoelectron take-off angle, and the angle between the X-ray source and the analyser was 54.7°. The high-resolution core level peaks were measured with a pass energy of 10 eV, in energy steps of 0.05 eV.

Operando X-ray Absorption Spectroscopy. The intensity of the Ni K fluorescence I_{fluo} (normalized to the incident photon flux) was collected at a fixed photon energy of 8345 eV, which corresponds to the point of maximum derivative of the x-ray absorption coefficient versus energy. This procedure maximizes the sensitivity to chemical shifts that induce a red- or blue-shift of the x-ray absorption edge. The operando measurements were conducted on the Ti:Fe_2O_3-NiMo and Ti:Fe_2O_3-Ni photoanodes immersed in the 0.1 M NaOH solution, both with and without HMF, as a function of the applied potential. The resulting plot of I_{fluo} vs applied potential E is named a Fixed Energy X-ray Absorption Voltammetries (FEXRAV). The negative derivative of I_{fluo} vs E, $-dI_{fluo}/dE$, or versus time $-dI_{fluo}/dt$ for constant potential sweeping rate, provides an element-selective analogue of the current recorded during a cyclic voltammetry. The experiments reported here were performed in absence of light stimuli, to avoid interference of the photoinduced carriers on the evaluation of the oxidation state of the cocatalyst as a function of the applied potential, assuming that the kinetics of the surface catalysis process is not affected by the nature of the carriers.

In addition, X-ray Absorption Near Edge Structure (XANES) spectra were recorded at fixed potentials. Comprehensive details of the experimental setup, including data collection at the LISA beamline at ESRF and the design of the custom 3D-printed PEC cell, are provided in *Section 3*.

Rate deconvolution procedure. The three-step rate deconvolution procedure was adapted from the methodology described by Bender et al.[4] and tailored to our study. Experiments were conducted using a flow-based one-compartment PEC cell containing 25 mL 0.1M NaOH solution (pH 13) and 6.6mM HMF. Two types of working electrodes were employed: Ti:Fe_2O_3-NiMo or NiMo sputtered on FTO. Hg/HgO (NaOH 1 M) was the reference electrode and Pt the counter electrode. The first, pre-conditioning step consisted of a chronoamperometry set at 1.4 V vs RHE for 30s during which the (photo)current density stabilized. For the Ti:Fe_2O_3-NiMo sample, this step was performed under continuous backside AM 1.5G illumination (100 $mW \cdot cm^{-2}$) to mimic the conditions used in photoelectrolysis experiments. Then, the Open Circuit Potential (OCP) was held for varying durations, immediately after switching off the light. Finally, the third step was performed by applying a reducing potential of 0.9 V vs RHE in the dark for 20s. This three-step procedure was repeated for each OCP duration (i.e., 0, 0.3, 0.7, 1, 1.5, 2, 3, 4… 10s), followed by kinetic analysis.

Prior to conducting the deconvolution experiments, CVs were performed on each sample at a scan rate of 20 mV/s to verify the (photo)electrochemical behaviour of each (photo)electrode in a 0.1 M NaOH solution (pH 13) w/wo 6.6 mM HMF.

1. SEM and PEC characterization

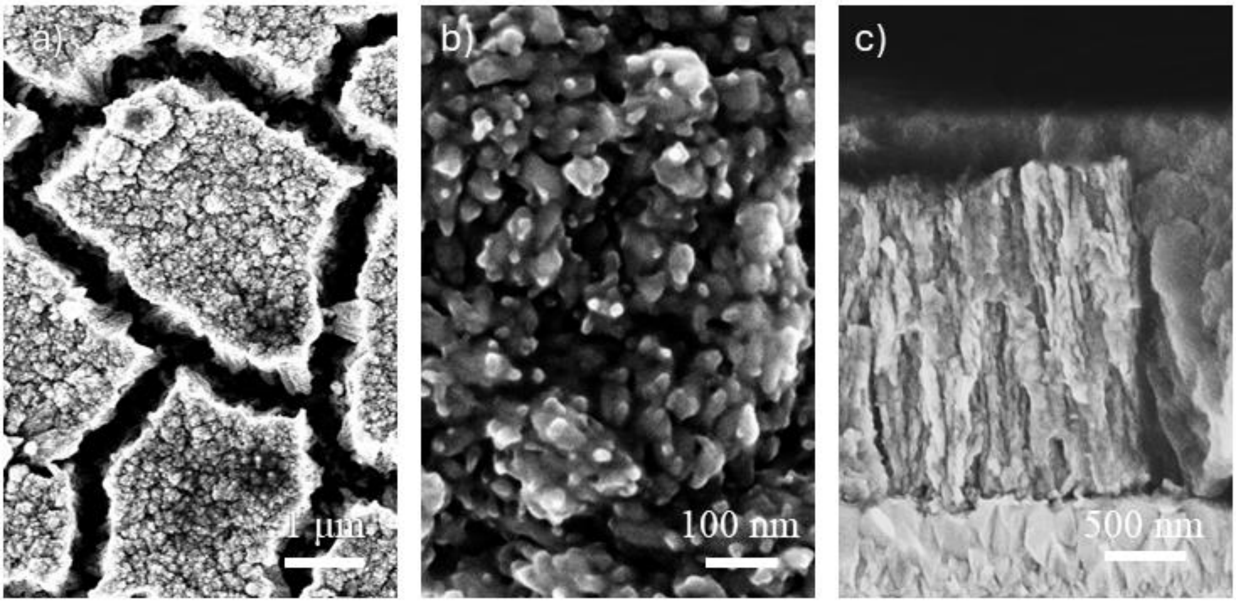

Fig. S1 (a,b) SEM Planar and **(c)** cross-sectional view of the Ti:Fe_2O_3 photoanodes.

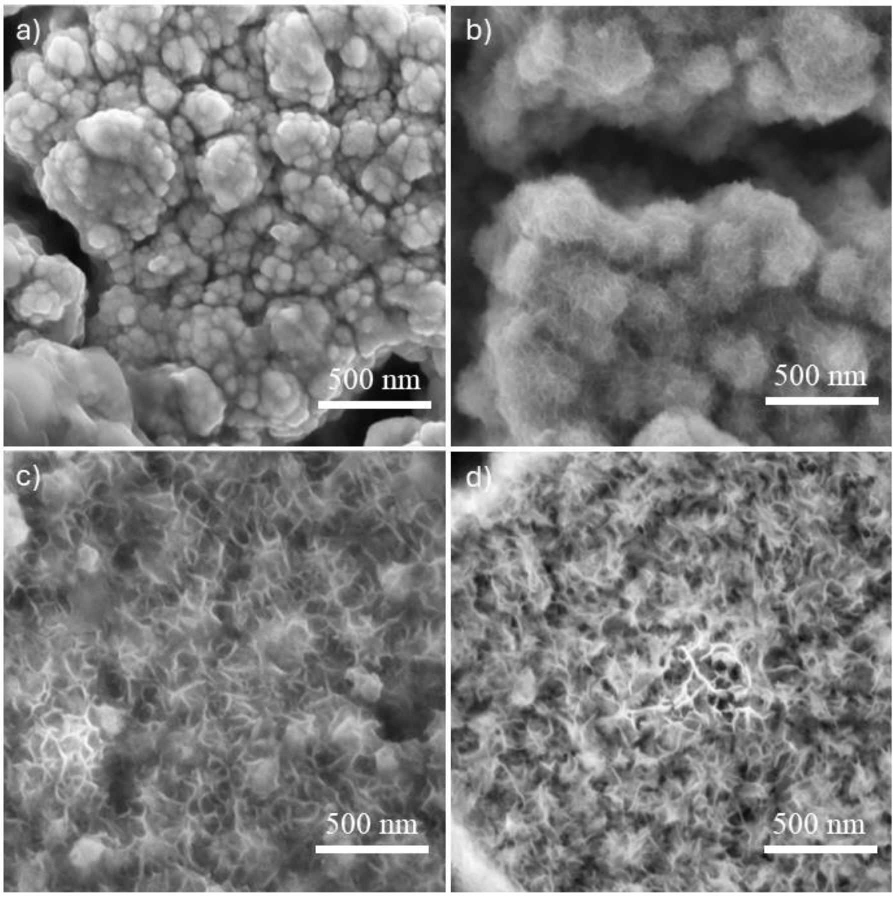

Fig. S2 SEM planar view of Ti:Fe_2O_3-NiMo before **(a)** and after **(b)** the HMF conversion experiment. **(c,d)** Analogue comparison for Ti:Fe_2O_3-Ni.

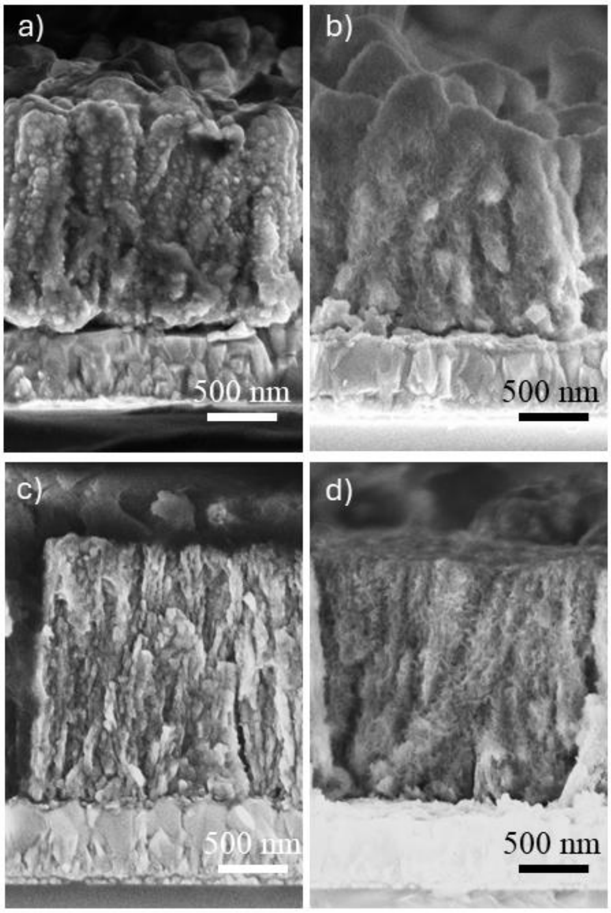

Fig. S3 SEM cross-sectional view of Ti:Fe_2O_3-NiMo before **(a)** and after **(b)** the HMF conversion experiment. **(c,d)** Analogue comparison for Ti:Fe_2O_3-Ni.

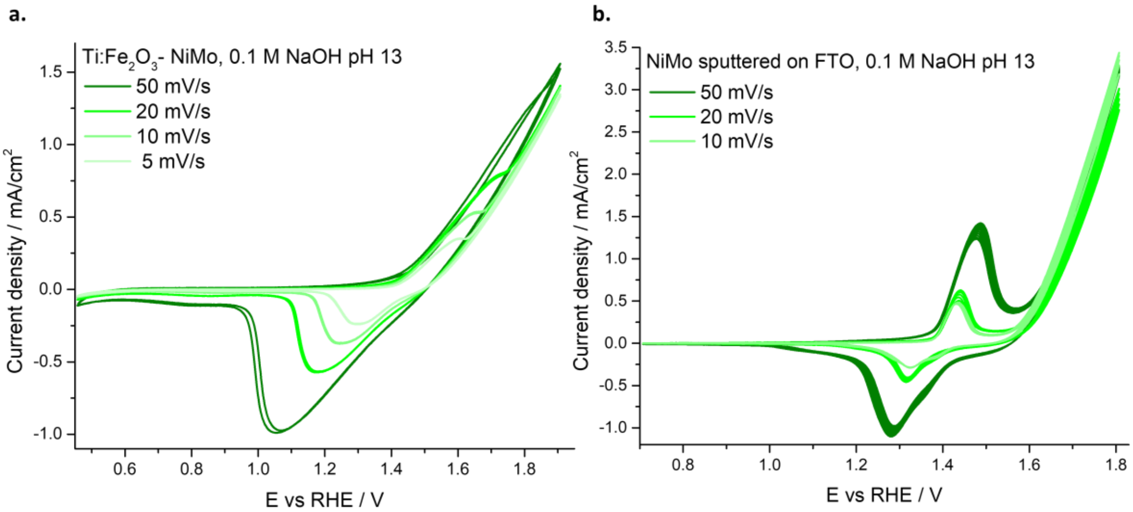

Fig. S4 Repeated dark CVs acquired for: (**a**) Ti:Fe_2O_3-NiMo photoanode; **(b)** NiMo-sputtered on FTO electrode. The brightening of the colours is related to the decreasing of the scan rate: from 50 mV/s to 5 mV/s and from 50 mV/s to 10 mV/s, respectively.

Investigation of the cathodic peak for Ti:Fe_2O_3-Ni photoanode. Cyclic voltammetry (CV) scans were performed in the dark under an argon atmosphere and compared to those in the air. The acquisition was done at different scan rates and in all cases the same peak at cathodic potentials was detected, indicating the reductive process was not related to oxygen reduction (Fig. S5a). To discard the potential influence of the underlying Ti-doped hematite, repeated dark CVs were acquired in air with nickel electrodeposited on

FTO at different scan rates (Fig. S5b). The extended cathodic feature persisted, pointing out its strong relation with $Ni(OH)_2$ electrocatalyst. In addition, as in the case of Ti:Fe_2O_3-NiMo, a linear dependence of the ox/red peak intensity on the scan rate was observed.

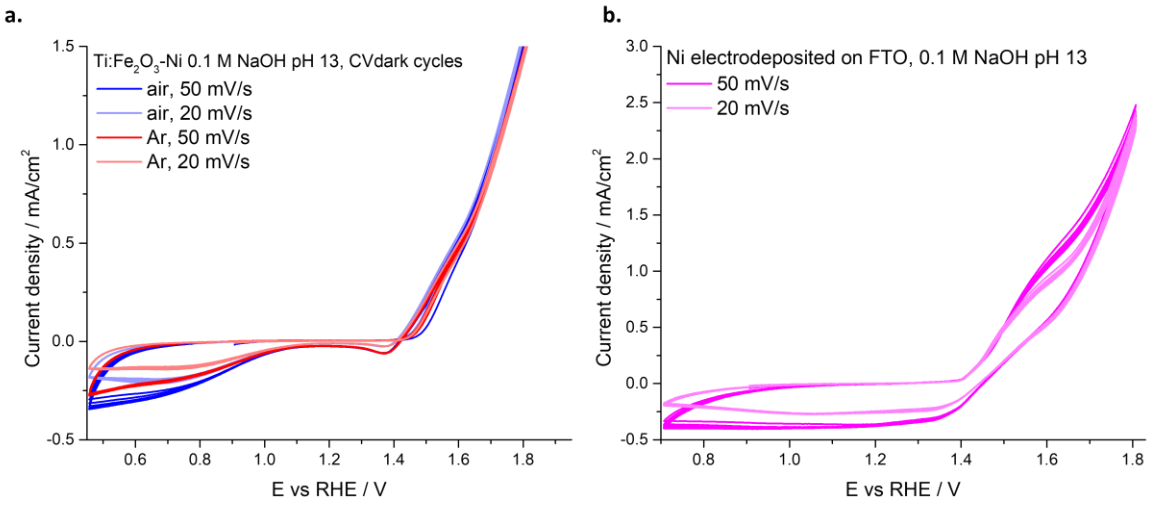

Fig. S5 (a) Comparison of repeated dark CVs acquired in air (blue lines) and in argon atmosphere (red lines) with Ti:Fe_2O_3- Ni photoanode; **(b)** Repeated dark CVs acquired in air with Ni-electrodeposited FTO (pink lines). In both cases, the brightening of the colours is related to the decreasing of the scan rate: from 50 mV/s to 20 mV/s.

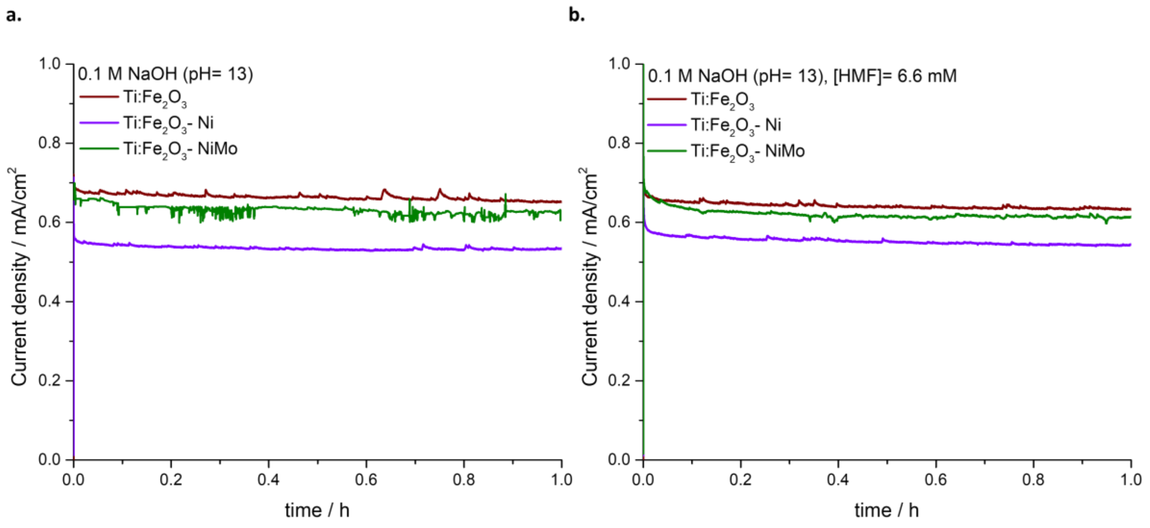

Fig. S6 Chronoamperometry for 1h at 1.4 V vs RHE, before **(a)** and after **(b)** the addition of HMF to the electrolyte. Ti:Fe_2O_3 (red), Ti:Fe_2O_3-Ni (green) and Ti:Fe_2O_3-NiMo (violet) photoanodes.

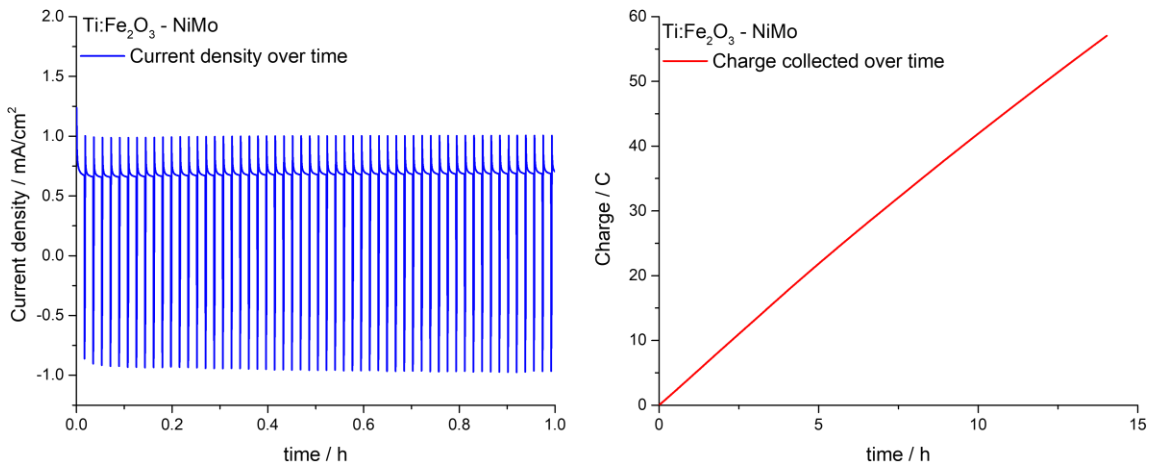

Fig. S7 Stability during long-term PEC HMF conversion. On the left: pulsed current density passed during the first hour; on the right: total charge accumulated throughout the duration of the measurement for Ti:Fe_2O_3-NiMo photoanode.

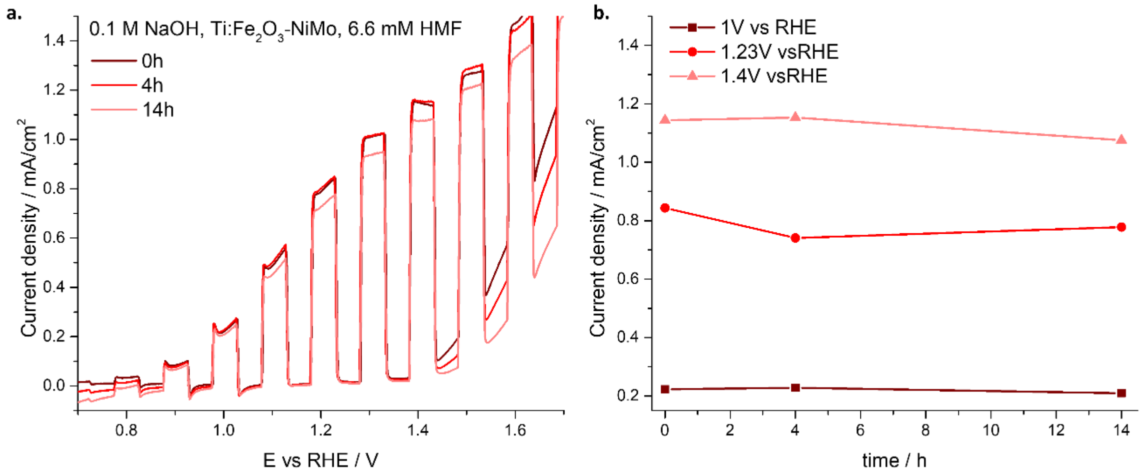

Fig. S8 (a) Evolution of the chopped LSVs for Ti:Fe_2O_3-NiMo photoanode during the HMF PEC conversion. **(b)** Corresponding trend of the photocurrent density over time at different potentials.

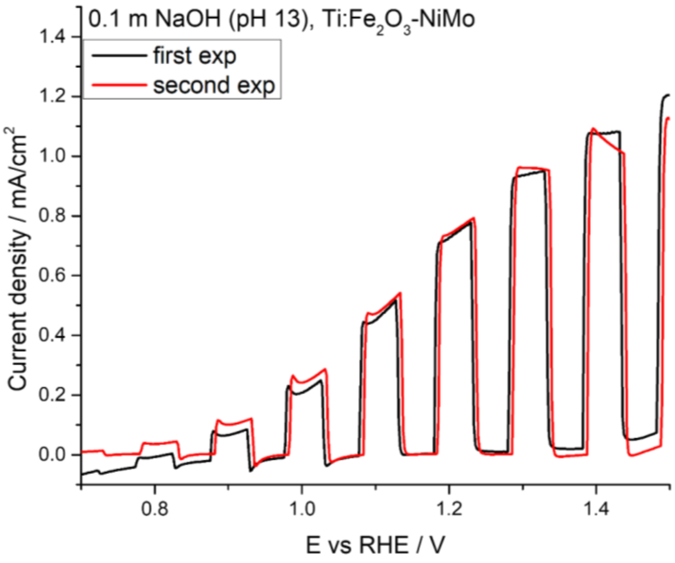

Fig. S9 Chopped LSVs for the same Ti:Fe_2O_3-NiMo photoanode acquired after two separated 6.6 mM HMF PEC conversion experiments.

2. Photoelectrochemical HMF conversion

Spectro-photometric characterization. The spectra of the standard compounds in water at 298 K: HMF, DFF, HMFCA, FFCA and FDCA were measured as reference (Fig. S10). The spectro-photometric analysis exploits the different shape of the absorption spectra of HMF and FDCA, with maxima at λ= 284 nm and λ= 263 nm, respectively. However, it does not allow to distinguish between HMF and FFCA as they have almost identical absorption spectra.

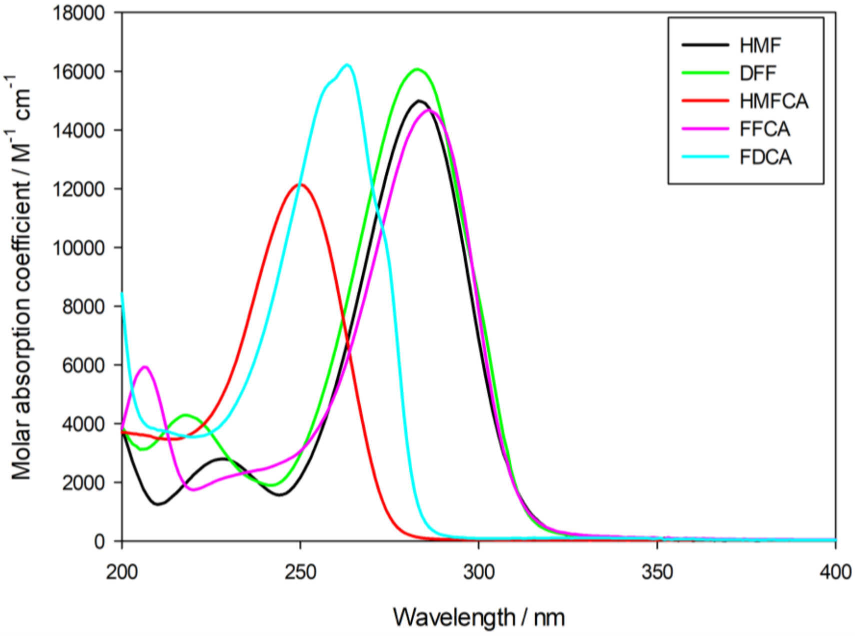

Fig. S10 Molar absorption coefficients of HMF and its oxidative intermediates as a function of the wavelength.

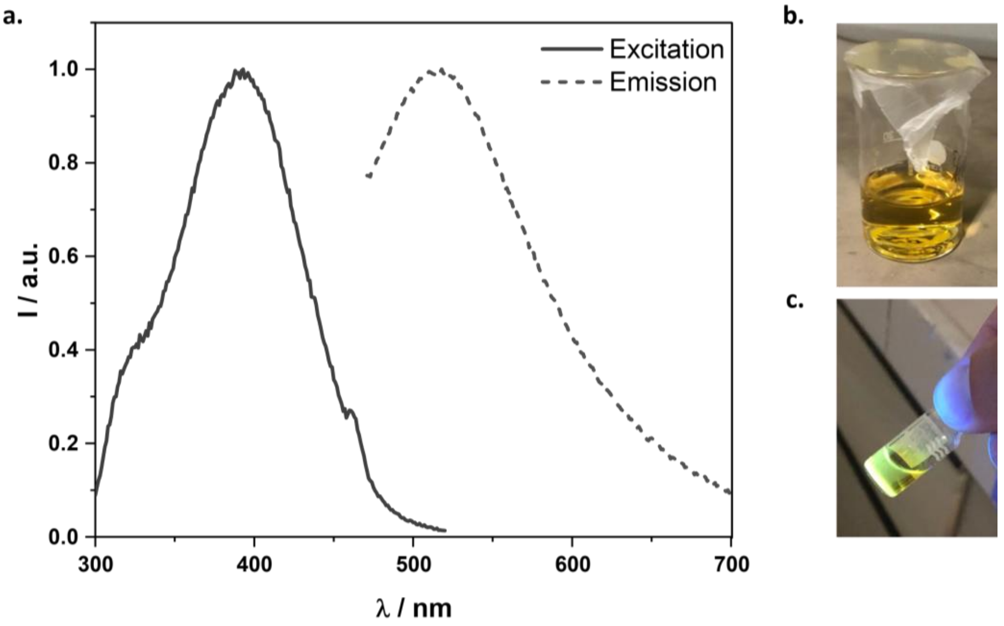

Fig. S11 (a) Excitation (λ_{em}= 550 nm, continuous line) and emission (λ_{exc}= 400 nm dotted line) spectra acquired after long-term PEC HMF conversion; **(b)** Colour of the anodic solution at the end of the experiment; **(c)** fluorescence of the solution.

Fitting equation. Since neither DFF nor HMFCA were detected by HPLC, the absorption spectra of the solution sampled at the start and at the end of the pulsed CA were fit by a linear combination of FFCA and FDCA reference spectra. Therefore, the fitting function was *ab= C1*a + C2*b*, where *ab* is the dependent variable, i.e. the final spectrum obtained out of the fitting; *a*, *b* are the independent variables, i.e. the absorption spectra of the standards considered; and *C1*, *C2* are the contributions of each standard to *ab*. In

particular, the coefficient *C1* quantifies the sum of FFCA and HMF, which is still present in the reaction mixture. The relative contribution of FDCA (%) to the final absorption spectra was calculated from the best fit coefficients according to the Equation:

$$\%FDCA_{fit} = \frac{C2}{C1 + C2} \text{x } 100 \qquad (S2)$$

Equation S3 showing the calculation of the FDCA relative conversion coefficient (%) from the HPLC data:

$$\%\ FDCA_{HPLC} \frac{FDCA\ conversion\ (\%)}{HMF\ residual +\ FDCA + FFCA\ (\%)} \text{x } 100 \qquad (S3)$$

where FDCA and FFCA conversion percentages (%) and HMF residual (%) are calculated from the HPLC spectra.

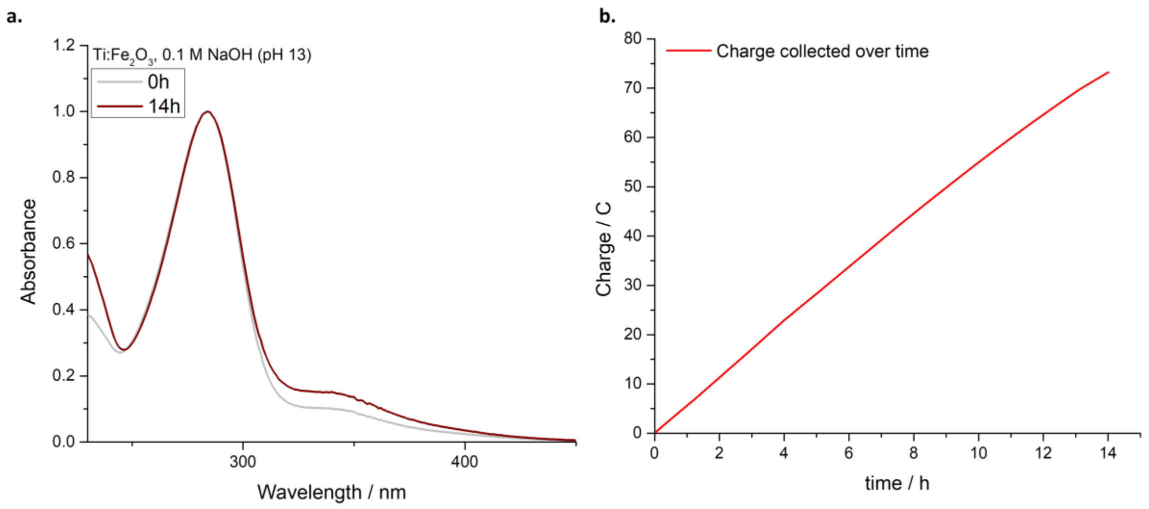

Fig. S12 Long-term PEC HMF conversion experiments with bare Ti:Fe_2O_3 photoanode. **(a)** Normalized absorption spectra; **(b)** charge collected during the chronoamperometry.

Investigation of the by-products and stability tests

Mo dissolution. The possible dissolution of Ni and NiMo cocatalysts under the applied bias was first examined, by repeating the chronoamperometry without the addition of HMF to the electrolyte. In this scenario, the solution remained transparent, and no absorbing species were detected (Fig. S13a). Via EDX analysis, the Ni/Fe and Ni/Mo ratio were determined and the results for Ti:Fe_2O_3-NiMo photoanode are shown in Fig. S13b. Interestingly, while the Ni/Fe ratio was maintained, the Ni/Mo ratio increased, suggesting a significant decrease in the Mo content after the chronoamperometry. This was further confirmed by the Mo 3d XPS spectra (Fig. S13c) and suggests the dissolution of Mo in basic aqueous environment.

a.

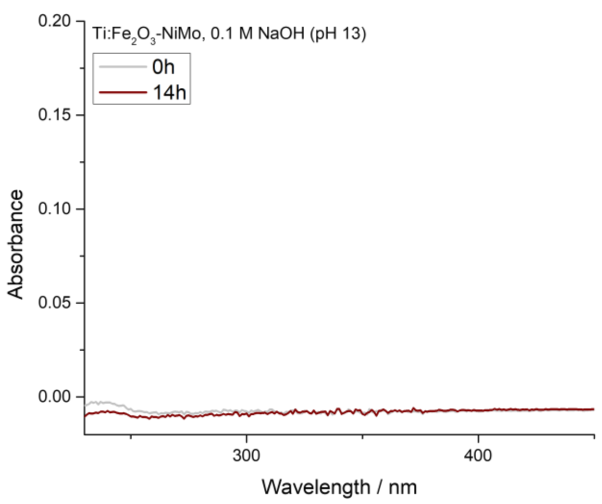

b.

Ti:Fe$_2$O$_3$- NiMo	Ni/Fe	Ni/Mo
Before CA	0.06	26.75
After CA	0.07	52

c.

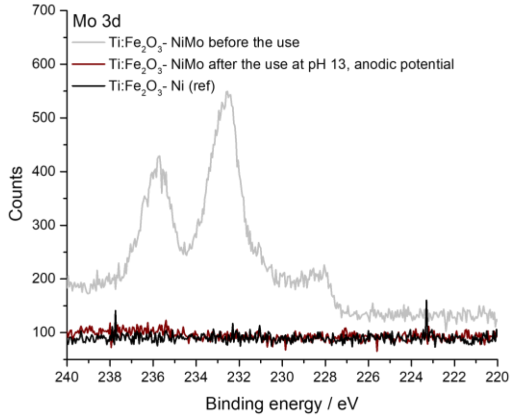

Fig. S13 Ti:Fe_2O_3-NiMo in a two compartments PEC cell without HMF, pulsed chronoamperometry (CA) at 1.4 V vs RHE. **(a)** absorption spectra at 0h (grey line) and at the end of the measurement (dark red line); **(b)** EDX analysis before and after the CA: Ni/Fe and Ni/Mo were calculated considering the Ni, Fe and Mo atomic percentages, by averaging them in different regions of the sample surface; **(c)** comparison of the Mo 3d XPS spectra for three different samples: Ti:Fe_2O_3-NiMo before the CA (grey line); Ti:Fe_2O_3-NiMo after the CA (dark red line) and Ti:Fe_2O_3-Ni taken as the reference (black line).

To check whether Mo dissolution alone may affect HMF conversion, an experiment was conducted by leaving Ti:Fe_2O_3-NiMo photoanode in 0.1 M NaOH electrolyte with 6.6 mM HMF, without applying bias and illumination. In this case, the HMF absorption spectrum did not change significantly throughout the experiment (Fig. S14). Therefore, we can safely rule out that the dissolution of Mo has any tangible effect on the observed HMF oxidation.

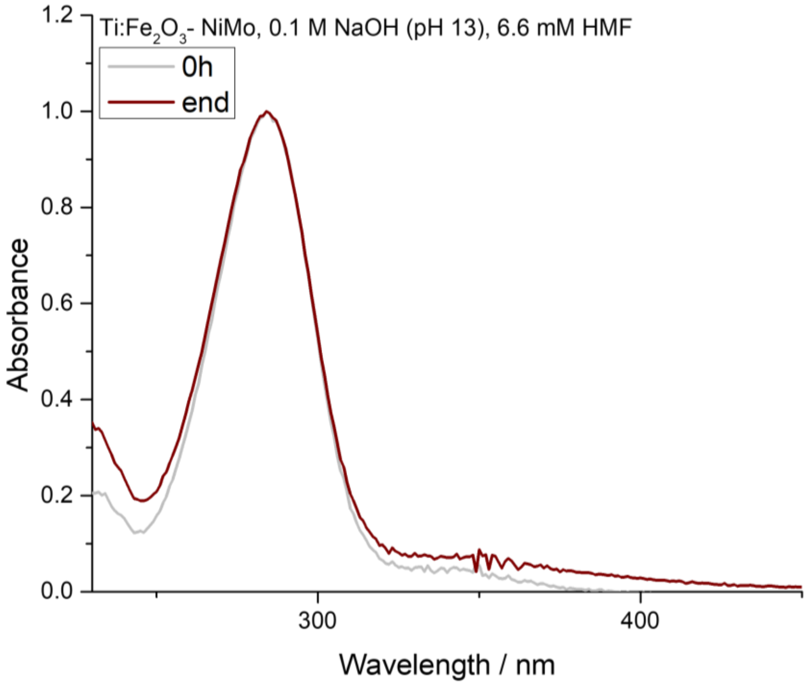

Fig. S14 Normalized absorption spectra, for no bias no light experiment with Ti:Fe_2O_3-NiMo photoanode and 6.6 mM HMF.

Stability tests. To investigate the stability of the solutions containing the biomasses, model samples with known concentration of HMF, DFF, FFCA or FDCA were prepared in the same conditions as the real samples (~mM in NaOH 0.1M) and stocked at 4°C. The stability of the samples in the experimental conditions was then evaluated *via* absorption spectra recorded at time intervals of 24h for 5 days. From this analysis, variation in the absorption spectra were appreciated only for HMF and DFF, whereas those of FDCA and FFCA were maintained. As widely reported in literature, these trials confirm the poor stability of HMF at high pH levels and demonstrate the durability of its oxidation product, FDCA, in basic conditions.

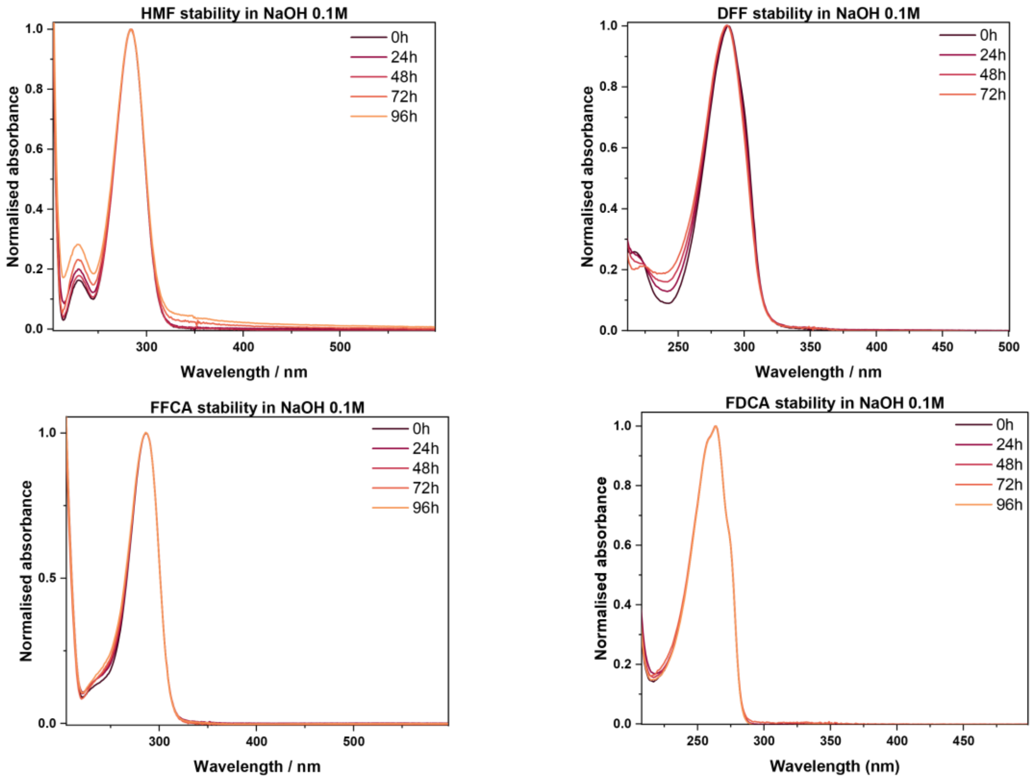

Fig. S15 Normalized absorbance of HMF, DFF, FFCA as a function of the wavelength. Stability tests performed in 0.1 M NaOH (pH 13), by acquiring absorption spectra at 0, 24, 48, 72 and 96 hours of the standards.

Additional photoelectrochemical HMF conversion experiments. To overcome the problem of HMF degradation, 6.6. mM HMF PEC conversion experiments were performed with Ti:Fe_2O_3-NiMo and Ti:Fe_2O_3-Ni photoanodes in different electrolytes, such as 0.25 M borate buffer and phosphate buffer, and at milder pHs 9 and 11.5. Pulsed CAs were conducted at potentials equal to 1.6 V and 1.3 V vs RHE, respectively. Samples were taken at the beginning and at the end of the measurements for a spectro-photometric product analysis. In all cases, no HMF conversion was observed, and no absorbing species were detected in the visible region of the spectra. Fig. S16 reports the results for Ti:Fe_2O_3-NiMo photoanode.

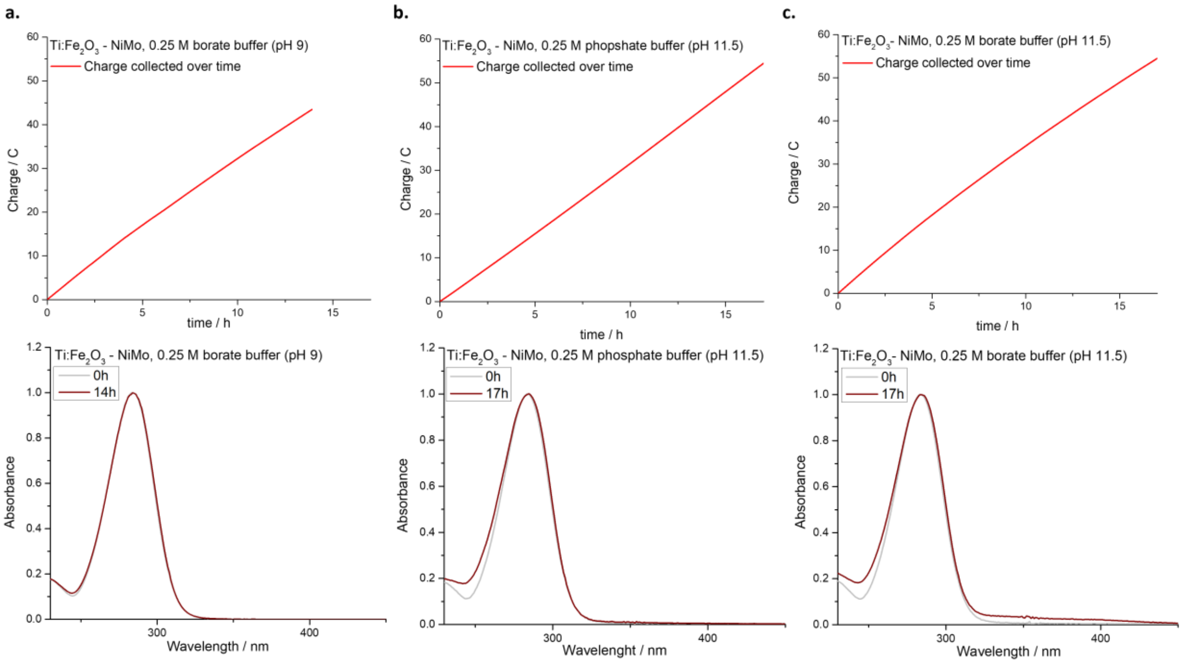

Fig. S16 On the top: charge collected during the pulsed CA; at the bottom: normalized absorption spectra. **(a)** CA at 1.6 V vs RHE in 0.25 M borate buffer (pH 9); CA at 1.3 V vs RHE in **(b)** 0.25 M phosphate buffer (pH 11.5); **(c)** 0.25 M borate buffer (pH 11.5).

3. Experimental procedure for operando x-ray absorption spectroscopy

X-ray absorption spectroscopy (XAS) was performed on the LISA BM-08 beamline at ESRF, Grenoble, using fluorescence mode. In order to perform operando measurements, we developed an experimental setup fully integrated with the beamline. The scheme is reported in Fig. S17a. This setup allows to control several experimental conditions for operando XAS directly from the control room of the beamline. The potential applied to the cell and the current can be monitored with the potentiostat, feeding this data directly to the acquisition system of the beamline for a precise synchronization between electrochemical and fluorescence data. In addition, thanks to the presence of a pulseless 3D printed peristaltic pump, the flux of the electrolyte in the cell can be controlled for enhancing mass transport and removing gaseous product from the electrode's surface. The 3D printed cell, Fig. S17b and c, was designed to minimize the thickness of the electrolyte in front of the sample (~100 µm) thereby reducing the attenuation of the X ray signal. This setup is compatible both with vacuum and with helium atmosphere in the main chamber.

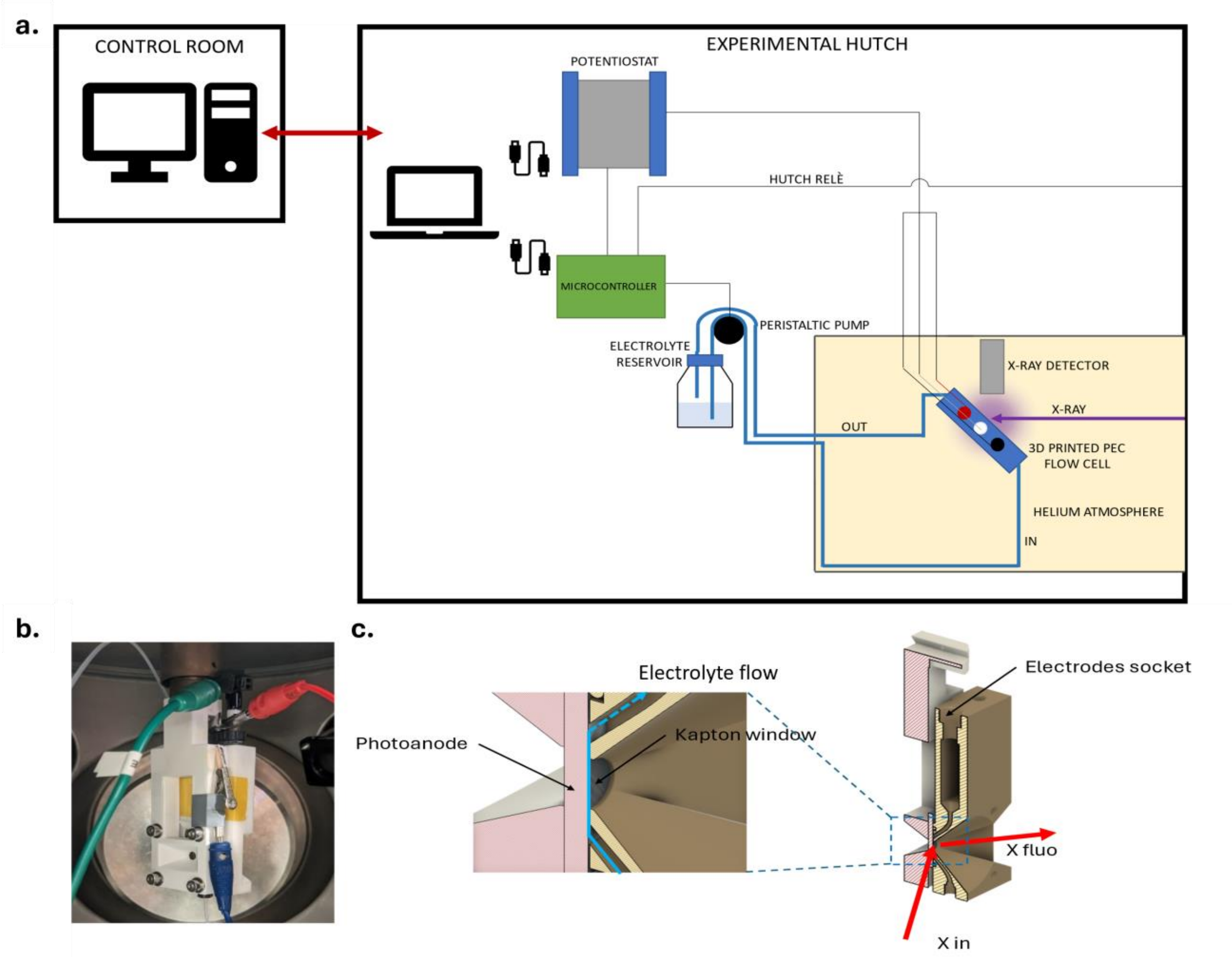

Fig. S17 Experimental setup developed for operando X-ray absorption measurements. **(a)** Scheme of the experimental setup with all the main components: potentiostat, microcontroller for pulseless peristaltic pump, electrolyte reservoir and 3D printed cell. **(b)** Picture of the cell in the vacuum chamber, **(c)** design of the cell, with cross-section of the cell on the left.

In Fig. S18, we report Fixed Energy X-ray Absorption Voltammetry (FEXRAV) for the two photoanodes. Ni K fluorescence intensity I_{fluo} (normalized to the incident flux) is recorded at a fixed incident photon energy of 8345 eV, corresponding to the maximum derivative of the Ni K-edge absorption spectrum. Two consecutive cyclic voltammetries were performed to check the stability of the process. The green line is the time derivative of the FEXRAV signal $-dI_{fluo}/dt$, which can be directly compared with current recorded by the potentiostat (blue line)[5].

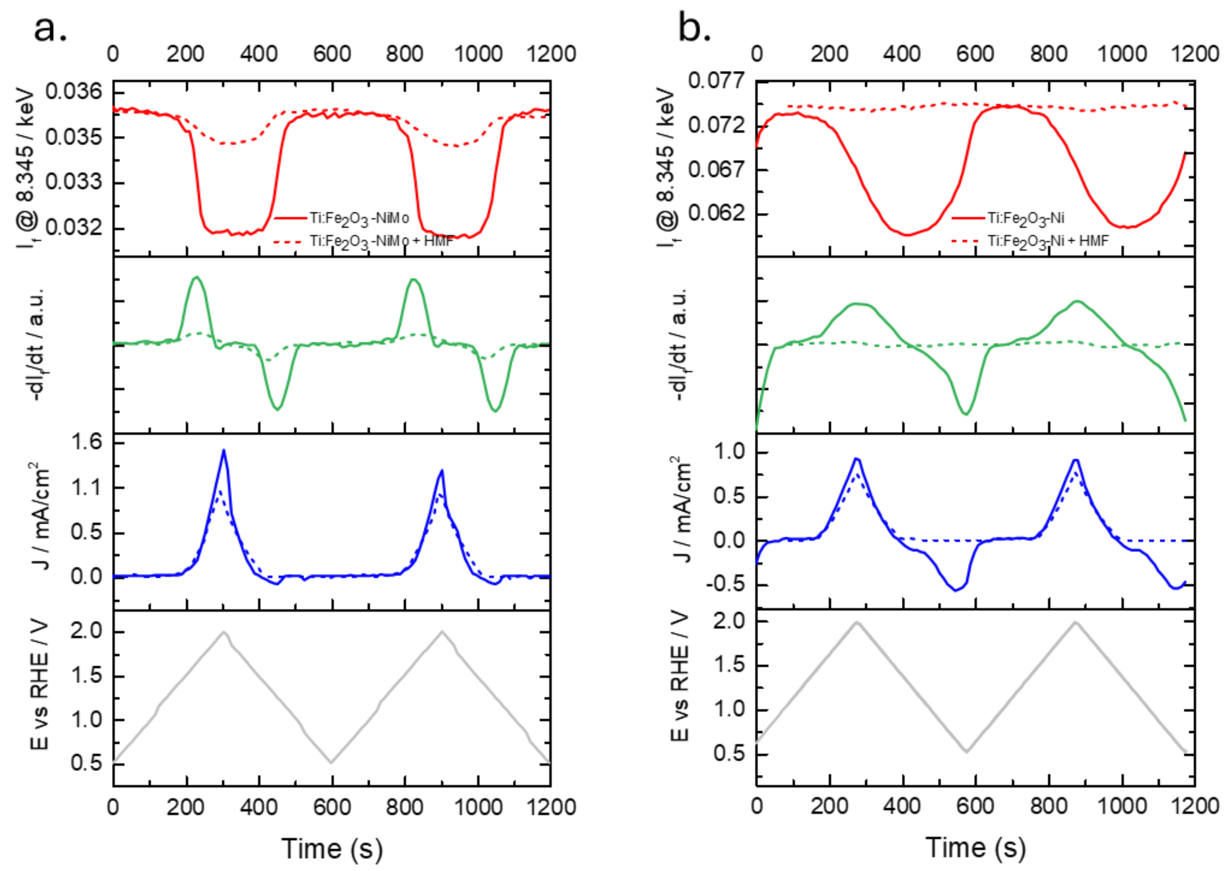

Fig. S18 FEXRAV signal recorded as a function of time during two consecutive cyclic voltammetries for **(a)** Ti:Fe_2O_3-NiMo and **(b)** Ti:Fe_2O_3-Ni (red line). For both samples, measurements were performed with and without the addition of HMF (dashed and continuous line, respectively). The green line is the derivative of the FEXRAV signal, to be directly compared to the current recorded by the potentiostat during the voltammetries (blue line).

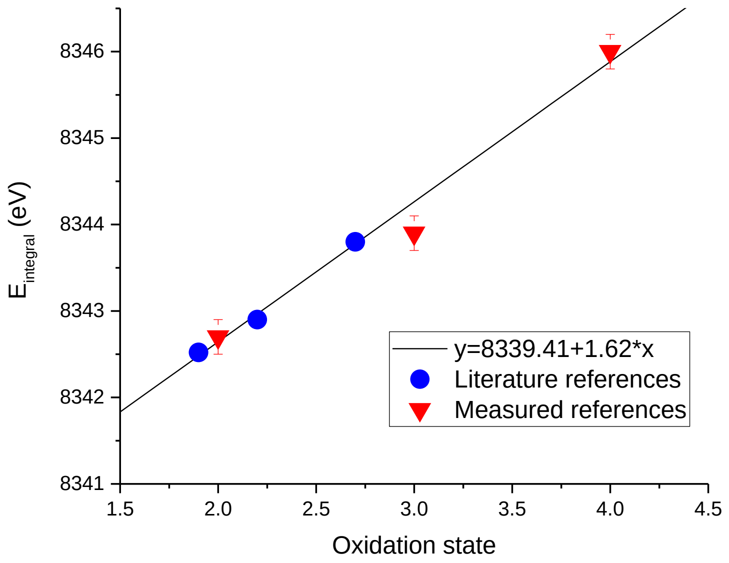

Fig. S19: Edge position by integration of the absorption edge for selected reference samples with linear calibration 1.62 ± 0.05 eV/Ox.State + 8339.41 ± 0.18 eV (red triangles). The used references were NiO, NiOOH, and $KNiIO_6$. Literature reference values for Ni-based electrocatalysts in operando conditions was reported for comparison[6].

4. Mechanistic discussion

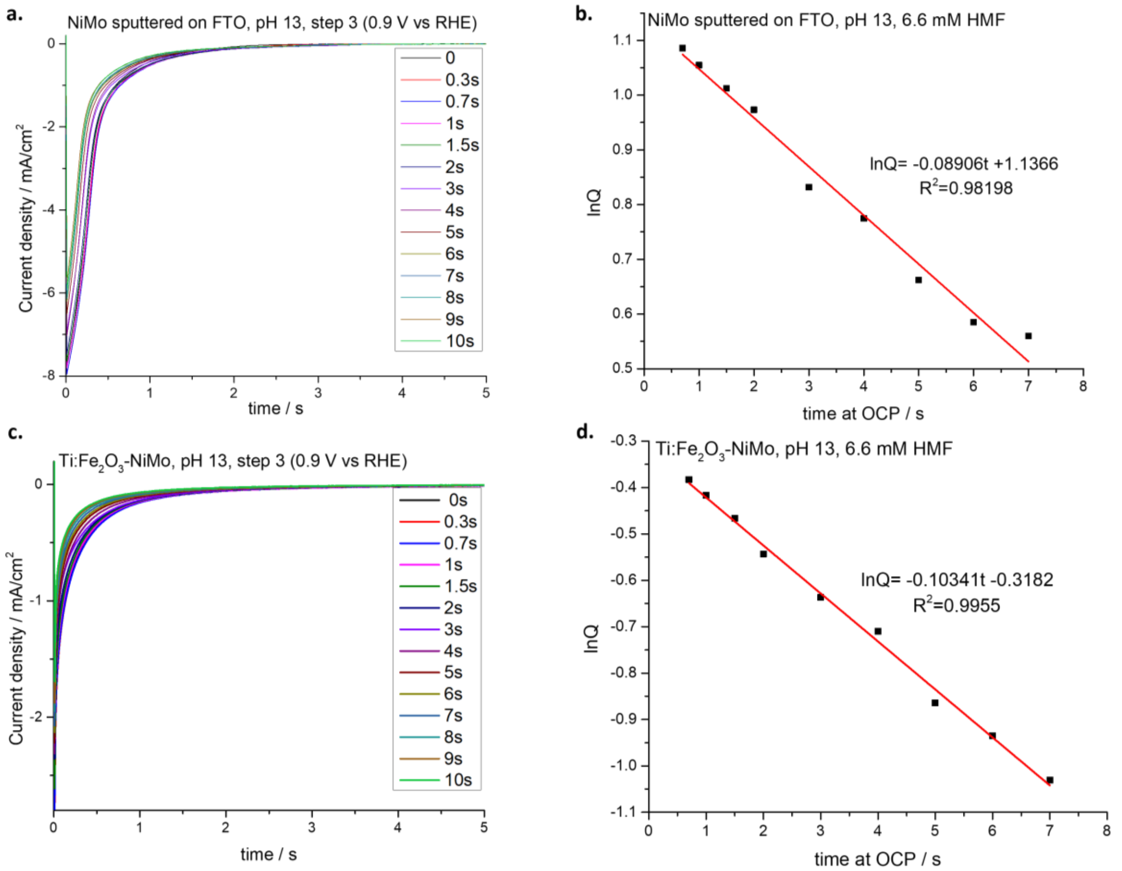

Fig. S20 (a,c) Chronoamperometries run at 0.9 V vs RHE to perform the reducing potential third step of the electroanalytical method. 0, 0.3, ..., 10s correspond to the time in which the system was left at OCP during the intermediate step of the rate deconvolution procedure; **(b,d)** Plots showing the disappearance of charge as a function of time from NiOOH films due to indirect oxidation of 6.6 mM HMF in a pH 13 solution under constant flow conditions.

As shown in Fig. S20b and d, the linearity of the ln(charge, Q) vs time plot indicates that the indirect oxidation of HMF can be modelled as a reaction with 1st order kinetics with respect to charge in the film. Thus, the rate of charge loss from the NiOOH film at time t is defined by the following pseudo first order rate law:

$$-\frac{dQ(t)}{dt} = k_{obs,ind} \cdot Q \ (S4)$$

Here, $k_{obs,ind}$ (s^{-1}) is obtained from the magnitude of the slope of the ln(charge) vs time plots, and Q is the charge stored in the NiOOH film at time t, i.e. the amount of charge required to reduce the film from its state at time t back to the $Ni(OH)_2$ initial state. After 0 seconds at open circuit ($t = 0$), i.e., immediately after the pre-conditioning constant potential step, the NiOOH film is exactly in the same state as it is during constant potential (photo)electrolysis. In this condition, the rate of charge loss from the film due to chemical oxidation matches the rate of the indirect process occurring during constant potential (photo)electrolysis. So, to determine I_{ind}, Equation S4 is solved for $t = 0$. The charge stored in the film at $t = 0$, denoted as Q_0, is obtained from the y-intercept of the ln(charge) vs time plot, as this represents lnQ_0. Once I_{ind} is known it can be compared to the steady-state current observed in step 1, I_{tot}, and used to calculate the value of I_{PD} according to equation:

$$I_{tot} = I_{ind} + I_{PD} \ (S5)$$

Table S1 shows calculation results for Ti:Fe_2O_3-NiMo and NiMo sputtered on FTO samples. The contribution of I_{ind} over I_{tot} is expressed as the ratio I_{ind}/I_{tot}. Notably, this value decreases tenfold for the Ti:Fe_2O_3-NiMo sample, due to the higher effective potential of the carriers photoinduced during step 1, in comparison to the ones produced in the dark with NiMo on FTO at the same applied bias, resulting in a major contribution of the PD mechanism to the overall process.

Sample	$k_{obs,ind}$ (s^{-1})	lnQ_0	Q_0 (mC/cm^2)	I_{ind} (mA/cm^2)	I_{tot} (mA/cm^2)	I_{PD} (mA/cm^2)	I_{ind}/I_{tot}
NiMo on FTO	0.0891	1.14	3.12	0.28	1.08	0.80	0.26
Ti:Fe_2O_3-NiMo	0.1304	-0.32	0.73	0.075	0.67	0.59	0.11

Table S1. Results from the three-steps rate deconvolution procedure applied for Ti:Fe_2O_3-NiMo and NiMo sputtered on FTO samples. $k_{obs,ind}$ (s^{-1}) and lnQ_0 are obtained, respectively, from the slope and the intercept of the lnQ vs time plots of Fig. S20b and d. The calculation of the following Q_0, I_{ind}, I_{tot}, I_{PD} and I_{ind}/I_{tot} parameters is explained above.

References

[1] D. K. Bora, *Mater. Sci. Semicond. Process.* **2015**, *31*, 728–738.

[2] J. Deng, J. Zhong, A. Pu, D. Zhang, M. Li, X. Sun, S.-T. Lee, *J. Appl. Phys.* **2012**, *112*, 084312.

[3] Y. Ling, G. Wang, D. A. Wheeler, J. Z. Zhang, Y. Li, *Nano Lett.* **2011**, *11*, 2119–2125.

[4] M. T. Bender, K. Choi, *ChemSusChem* **2022**, *15*, 1–14.

[5] R. D. L. Smith, C. Pasquini, S. Loos, P. Chernev, K. Klingan, P. Kubella, M. R. Mohammadi, D. Gonzalez-Flores, H. Dau, *Nat. Commun.* **2017**, *8*, 2022.

[6] D. González-Flores, K. Klingan, P. Chernev, S. Loos, M. R. Mohammadi, C. Pasquini, P. Kubella, I. Zaharieva, R. D. L. Smith, H. Dau, *Sustain. Energy Fuels* **2018**, *2*, 1986–1994.
